# Phase II Study of Cabozantinib in Patients With Bone Metastasis

**DOI:** 10.1093/oncolo/oyac083

**Published:** 2022-05-07

**Authors:** Edwin Choy, Gregory M Cote, M Dror Michaelson, Lori Wirth, Justin F Gainor, Alona Muzikansky, Lecia V Sequist, Ryan J Sullivan, Panagiotis M Fidias, Alice Shaw, Rebecca S Heist

**Affiliations:** Division of Hematology Oncology, Massachusetts General Hospital, Massachusetts General Hospital Cancer Center, Boston, MA, USA; Division of Hematology Oncology, Massachusetts General Hospital, Massachusetts General Hospital Cancer Center, Boston, MA, USA; Division of Hematology Oncology, Massachusetts General Hospital, Massachusetts General Hospital Cancer Center, Boston, MA, USA; Division of Hematology Oncology, Massachusetts General Hospital, Massachusetts General Hospital Cancer Center, Boston, MA, USA; Division of Hematology Oncology, Massachusetts General Hospital, Massachusetts General Hospital Cancer Center, Boston, MA, USA; Massachusetts General Hospital Biostatistics Center, Boston, MA, USA; Massachusetts General Hospital Cancer Center, Boston, MA, USA; Division of Hematology Oncology, Massachusetts General Hospital, Massachusetts General Hospital Cancer Center, Boston, MA, USA; Division of Hematology Oncology, Massachusetts General Hospital, Massachusetts General Hospital Cancer Center, Boston, MA, USA; Division of Hematology Oncology, Massachusetts General Hospital, Massachusetts General Hospital Cancer Center, Boston, MA, USA; Medical Oncology, Center for Cancer Care, Exeter Hospital, Exeter, NH, USA; Division of Hematology Oncology, Massachusetts General Hospital, Massachusetts General Hospital Cancer Center, Boston, MA, USA; Novartis Institutes for BioMedical Research, Cambridge, MA, USA; Division of Hematology Oncology, Massachusetts General Hospital, Massachusetts General Hospital Cancer Center, Boston, MA, USA

**Keywords:** bone metastasis, cabozantinib, sarcoma, skeletal-related events, MET and VEGF inhibition

## Abstract

Bone metastases are often difficult to manage as they can be symptomatic and skeletal-related events (SREs) can contribute to significant morbidity and declines in performance status. We sought to identify a novel medical treatment for bone metastasis by testing the safety and efficacy of cabozantinib in patients with bone metastasis arising from non-breast, non-prostate, malignant solid tumors. Patients were administered cabozantinib as an oral drug starting at 60 mg per day and radiologic measurements were performed at baseline and every 8 weeks. Thirty-seven patients were enrolled. No SREs were observed throughout the study. Twenty patients had disease measurable by Response Evaluation Criteria in Solid Tumors (RECIST) version 1.1. Four of 20 had a partial response by RECIST. An additional 12 patients had some decrease in tumor burden with nine of these having a decrease in tumor burden of at least 10% by RECIST. Six of the patients with at least a minor response had sarcoma. Sixteen patients had biomarkers of bone turnover measured before and after treatment. Most of these patients demonstrated decrease in urine and serum N-telopeptide and serum C-telopeptide. However, these changes in biomarkers of bone turnover did not correlate with radiographic changes measured by RECIST. This study demonstrates clinical activity and safety for cabozantinib in heavily pretreated patients with bone metastasis and shows activity for cabozantinib in patients with metastatic sarcoma.

## Teaser

Bone metastases are often difficult to manage. This article reports on the clinical activity and safety of cabozantinib in patients with bone metastasis and reports activity for cabozantinib in patients with metastatic sarcoma and other cancers.

**Implications for Practice**

This is an open-labeled phase II study of cabozantinib performed at Massachusetts General Hospital. The study population included patients with non-breast and non-prostate cancer with bone metastasis. The investigators observed reduction in visceral metastasis in many patients who participated in this study. For some, the duration of response proved to also be quite significant. Most of the patients with sarcoma who participated in this study experienced some decrease in tumor dimensions as determined by Response Evaluation Criteria in Solid Tumors. These observations are important in providing data in support of further studying cabozantinib in sarcomas and possibly other cancers as well.

## Introduction

The skeleton is a common site of metastasis for many cancers. Bone metastases are challenging to manage as they are often quite morbid and symptomatic. Skeletal-related events (SREs) from bone metastases include pathologic fracture, which leads to substantial pain and disability, vertebral compression, and hypercalcemia. Although radiation and surgery are often used to manage symptomatic bone metastasis, patients with multiple lesions are often not suitable candidates for these procedures. Much research has already been devoted to understanding how to reduce SREs in patients with metastatic breast and prostate cancer using osteoclast inhibitors. Denosumab, which inhibits the RANKL signaling pathway, and zoledronic acid, a type of bisphosphonate, were both approved by the US Food and Drug Administration (FDA) for risk reduction from SREs in patients with bone metastasis. Other signaling pathways, such as mesenchymal epithelial transition factor (MET) and vascular endothelial growth factor (VEGF) pathways, can play important roles in osteoblast and osteoclast function,^1-4^ suggesting a role for inhibition of these pathways in patients with bone metastasis. MET and VEGF inhibition may also abrogate tumor proliferation.

MET is a receptor for hepatocyte growth factor (HGF) with downstream signaling pathways affecting tumor survival, growth, angiogenesis, invasion, and dissemination.^5-7^ Inhibition of MET demonstrated anti-tumor activity in phase I trials.^8-13^ Vascular endothelial growth factor receptor (VEGFR) is a mediator of angiogenesis in tumors, and inhibition of VEGF/VEGFR pathway has shown survival benefit in colon cancer^14,15^ and lung cancer.^16-18^ Drugs that simultaneously inhibit MET and VEGFR may have more anti-tumor activity than agents that target only one of these tyrosine kinase receptors.^7,19^ Animal studies suggest inhibition of MET and VEGFR-2 may be synergistic.^1,19-25^

Cabozantinib (XL-184, Cabometyx), which inhibits MET, rearranged during transfection (RET), and VEGFR-2,^26^ has been shown to block both osteoblastic and osteolytic progression of xenograft tumors in bone,^2-4^ suggesting potential clinical activity in patients with bone metastasis. Cabozantinib was granted FDA approval in 2011 to be marketed as treatment for progressive metastatic medullary thyroid cancer^27^ and in 2016 for treatment of advanced renal cell carcinoma.^28^ It is also being developed as a therapy against other cancers that express MET, VEGFR-2, and RET.^29-32^

Despite the importance of bone metastases for both morbidity and mortality from advanced solid tumors, there is no clear consensus on how best to measure response to cancer therapeutics in bone. Bone metastases are considered “unmeasurable” by standard Response Evaluation Criteria in Solid Tumors (RECIST) criteria, and indeed there is no consensus on the optimal imaging modality for evaluating bone metastases.^33^ Biomarkers of bone activity may be a useful surrogate to measure response in bone metastases, and many clinical trials have used these as surrogate endpoints.^34,35^ Urinary N-telopeptide (Ntx), serum Ntx, and serum C-telopeptide (Ctx), among others, have been studied as biomarkers of bone metabolic activity.,^36-38^. However, it is not well understood which biomarker best measures metabolic bone turnover in correlating with clinical outcomes.

We designed a phase II clinical trial to test the safety and efficacy of cabozantinib in patients with solid tumor (non-breast, non-prostate) cancers and bone metastasis (ClinicalTrials.gov Identifier: NCT01588821). Additionally, we sought to measure bone urinary and serum Ntx and serum Ctx to determine if they can serve as biomarkers for tumor activity in bone metastasis.

## Methods

Patients were recruited for this study if they were diagnosed with a solid tumor malignancy other than breast or prostate cancer, were older than 18 years old, and had bone metastasis. Inclusion criteria required metastatic disease that was refractory to or relapsed/progressed after standard therapies. The presence of bone metastasis was required. Eastern Cooperative Oncology Group performance status had to be 0 or 1. Organ and marrow function had to be intact, including absolute neutrophil count (ANC) ≥ 1500/mm^3^ without colony-stimulating factor support; Platelets > 100 000/mm^3^; Hgb > 9 g/dL; Bilirubin ≤ 1.5 × the upper limit of normal (ULN); serum creatinine ≤ 1.5 × ULN or creatinine clearance ≥ 50 mL/minute; alanine transaminase (ALT) and aspartate transaminase (AST) ≤ 2.5 × ULN if no liver involvement; or ≤ 5 × ULN with liver involvement. Women of childbearing age must have a negative pregnancy test at screening and agree to strict adherence to contraception during the course of the study and for 4 months after the last dose of study drugs. Granulocyte colony stimulating factor (GCSF) support was not allowed in study. Patients were excluded if they had an active infection, brain metastasis, brain tumor, or epidural disease. Other standard exclusion criteria for clinical trials are listed in the protocol available in the Supplementary Material.

Patients were consented and enrolled in this IRB-approved, open-label phase II study at Massachusetts General Hospital (Protocol 12-091). This study was posted on clinicaltrials.gov as NCT01588821.

The starting dose was 60 mg per day of cabozantinib, taken orally, swallowed intact with a minimum of 8 oz water, without dissolving or crushing, after fasting for 2 h. Missed or vomited doses were not allowed to be replaced. Dose reductions at 20 mg intervals or interruptions were applied for intolerable grade 2 or higher adverse events that were determined to be related to cabozantinib. See protocol in the Supplementary Material for complete details.

Computed Tomography scans of the tumor, chest, abdomen, and pelvis were performed at baseline and repeated every 2 cycles (each cycle = 28 days) until disease progression. Radiologic tumor responses were evaluated by RECIST version 1.1. Progression-free survival (PFS) timepoints were based on the date of study enrollment and the date of scans showing disease progression or the date of coming off study for clinical decline. Median PFS and PFS timepoint estimates were calculated based on the Kaplan-Meier method (KM). Urine and serum Ntx were taken along with other laboratory measurements at baseline during the study screening process and at week 8 of the study. Greater than 40% decrease in urinary Ntx, serum Ntx, or serum Ctx at week 8 were determined to represent biomarker response to treatment.

A 2-stage phase II design was used, with an interim analysis and an early stopping rule for inactivity. The first stage planned to include 28 evaluable patients by bone biomarker results. If at least 5 of 28 evaluable patients achieve a bone biomarker response to treatment, defined as ≥ 40% decrease in urinary Ntx, serum Ntx, or serum Ctx at week 8, enrollment will proceed with 10 additional patients. The underlying assumption is that the regimen will be of interest if the proportion of patients achieving the > 40% decrease in bone biomarker is ≥ 45%, and not of interest if the proportion achieving the endpoint is ≤ 20%. This design aimed for an overall significance level of 0.05 with power of 90%. One of the 10 patients in the expansion cohort was enrolled but did not initiate treatment on study, so they were excluded from analysis.

## Results

Thirty-seven patients enrolled in this study (Table 1). Fourteen had sarcoma, 7 had renal cell carcinoma, 5 had non–small cell lung cancer (2 with RET rearrangement), three had head and neck carcinoma (squamous cell carcinoma, salivary duct carcinoma, and olfactory neuroblastoma), 3 had radioiodine-resistant differentiated thyroid cancer, 2 had melanoma, one had adenoid cystic carcinoma, one had metastatic chondroblastoma, and one had chordoma. This cohort of patients was heavily pretreated with prior chemotherapy (average lines of therapy: 2.5; range: 0-9). Patient age at enrollment ranged from 18 to 83 years, with an average age of 54 years old. Thirty-seven percent of patients were female.

**Table 1.** Baseline characteristics.

Baseline characteristics	*N*
Number of patients	37
Median age, years (range)	54 (18-83)
Gender	
Male	23
Female	14
No. of patients with RECIST measurable disease	20
No. of patients with bone-only disease	17
Cancer type	
Renal cell	7
Lung non-small	5
Osteosarcoma	3
Radioiodine-refractory differentiated thyroid cancer	3
Ewing's sarcoma	3
Chondrosarcoma	2
Leiomyosarcoma	2
Melanoma	2
Alveolar soft parts sarcoma	1
Head and neck squamous cell carcinoma	1
Adenoid cystic carcinoma	1
Chondroblastoma	1
Chordoma	1
Fibroblastic sarcoma	1
Liposarcoma	1
Myxofibrosarcoma	1
Salivary duct carcinoma	1
Olfactory neuroblastoma	1

Abbreviations: RECIST, Response Evaluation Criteria in Solid Tumors.

The vast majority of participants did not use bone-targeted agents prior to the study, and no participant used bone-targeted agents during the study. Only 4 of 37 participants used bisphosphonates prior to the study and only one participant used denosumab prior to the study. Two other participants used bisphosphonates only after completion of the study. Because no participants used bone modifying agents during the study, we did not have to account for their use to interpret bone biomarker changes.

**Figure 1.**
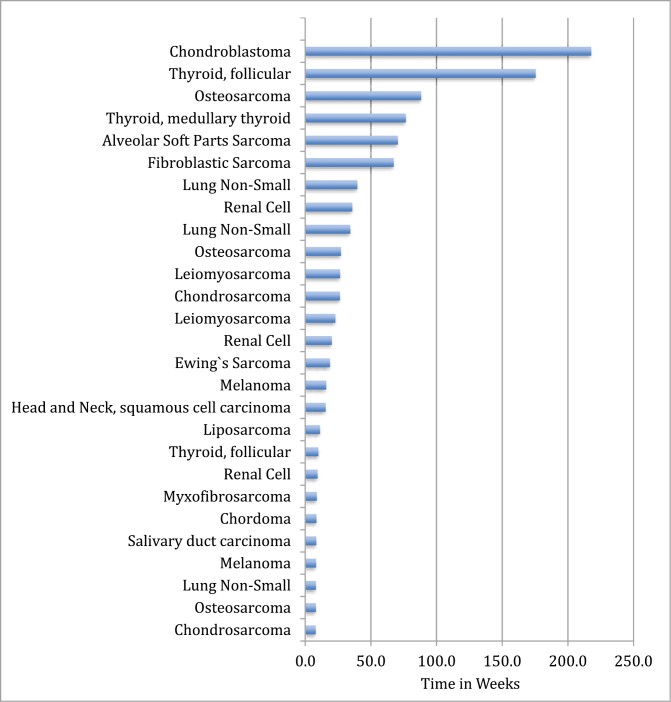
Time to disease progression (weeks).

**Figure 2.**
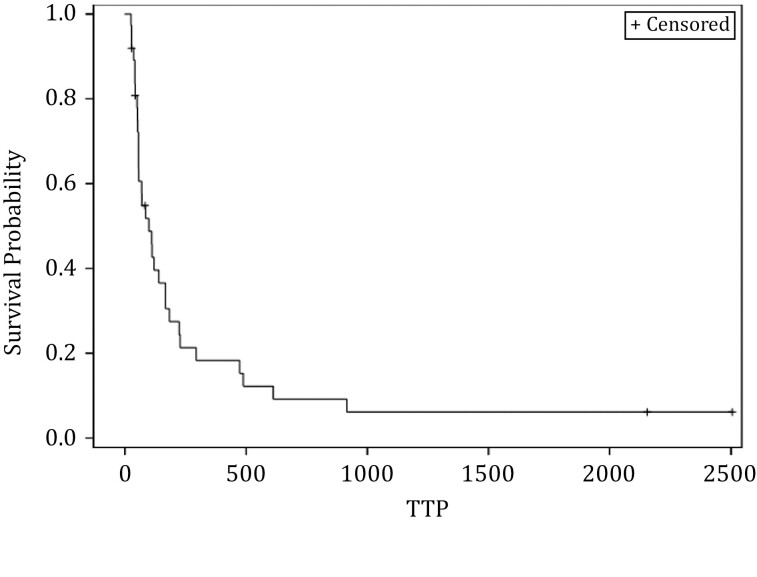
Kaplan-Meier plot of time to disease progression (days).

**Figure 3.**
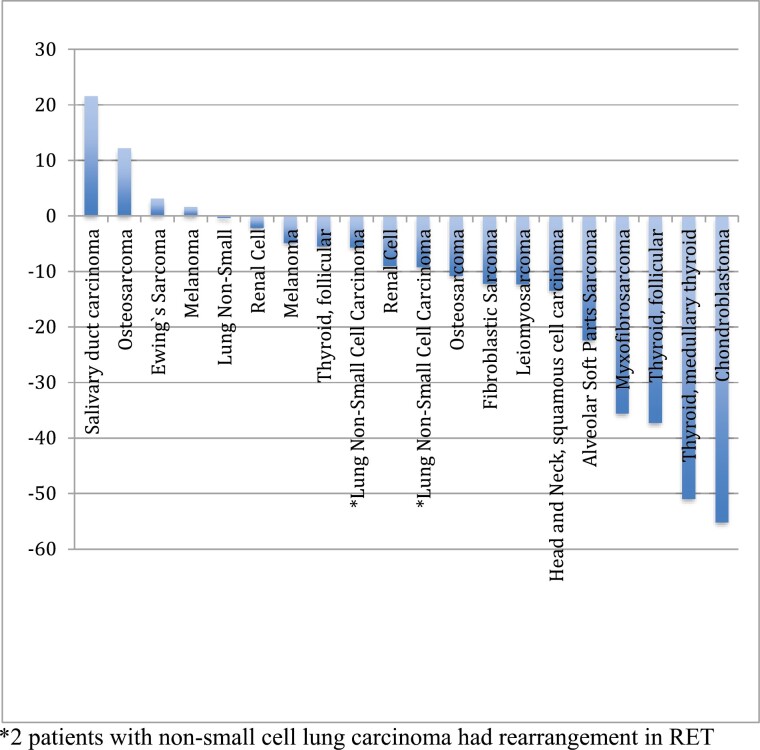
Waterfall plot of percentage of best responses.

Figure 1 shows time to disease progression for each of the 27 patients for which this data was available. The median PFS was 3.5 months, 6-month PFS was 30%, and 1-year PFS was 18% (Fig. 2). Among the 20 patients with disease measurable by RECIST (the others had bone-only disease, which does not qualify for RECIST evaluation), 4 patients had a partial response (Fig. 3). These patients had chondroblastoma, myxofibrosarcoma, and radioiodine-resistant differentiated thyroid cancer. Sixteen had at least a minimal decrease in the tumor as best response, and 9 of those 16 had a decrease in tumor size by 10% or greater by RECIST. Among the 9 patients who had a decrease in tumor size of at least 10%, six had sarcoma and 2 had thyroid cancer. The other 17 of 37 enrolled patients had bone-only disease and were not measurable by RECIST.

Two of 3 patients with thyroid cancer experienced a partial response by RECIST tumor measurements. One of two patients with osteosarcoma experienced a 10.8% decrease by RECIST. This patient remained on study for 20 weeks when she ultimately developed disease progression. The other patient with osteosarcoma had SD as her best response, but fluorodeoxyglucose (FDG) uptake decreased and detectable areas of prior Tc99m uptake completely resolved. She remained on study for 87 weeks until disease progression. A patient with leiomyosarcoma had 12.3% decrease in RECIST measurements as best response, but his tumor demonstrated decreased FDG and Tc99m in most lesions while on study drug. He experienced disease progression after 24 weeks. A patient with fibroblastic sarcoma had 12.2% decrease in RECIST measurements as her best response. FDG uptake of her lesions were unchanged and Tc99m uptake of her lesions decreased. She remained on study for 67 weeks until disease progression. The patient with chondroblastoma remains in partial response (PR) at the date of this manuscript submission (in the KM analysis, this patient was censored for coming off study to move to Florida). All of these patients described in the vignettes above surpassed the 7-week average PFS seen in patients with metastatic soft-tissue sarcoma.^39^ Of the 13 patients in this study with sarcoma and measurable disease (not including the patient with chondroblastoma), the average PFS was 30 weeks. Most of the disease progression was in the lungs or abdominal viscera. Only 6 patients progressed in bone as their first site of progression.

Overall, treatment with cabozantinib in this cohort was tolerable, but 55% of patients required dose reductions for toxicities. The most common adverse events were fatigue and diarrhea. Table 2 lists toxicities that were at least possibly attributed to cabozantinib with a frequency of > 10% among patients. No grade 4 toxicities were observed. No SREs were observed during this study.

**Table 2.** adverse events (included if >10% in frequency and at least possibly attributed to cabozantinib).

Toxicity/grade	1	2	3
Fatigue	18	15	2
Diarrhea	16	9	0
Elvated AST	18	1	0
Palmar-plantar erythrodysesthesia	10	6	0
Anorexia	11	4	1
Nausea	6	7	1
Weight loss	6	6	1
Phosphate decreased	4	5	4
Hypertension	6	5	1
Elevated ALT	10	1	0
Lipase elevated	5	0	3
Platelets decreased	7	1	0
Anemia	5	1	1
Alkaline phosphatase elevated	7	0	0
Malaise	4	1	0
Hypothyroidism	1	3	0
GE reflux	4	0	0
Oral dysesthesia	4	0	0
Vomiting	4	0	0
Serum amylase increased	4	0	0
Dysgeusia	4	0	0
QTc prolonged	0	0	1

QTc is the distance between Q and T waves in ECG, corrected by heart rate.

Serum and urine Ntx and serum Ctx were obtained with other protocol lab draws at screening and at week 8 of the trial. Unfortunately, after bone biomarkers were collected on 16 patients, the lab performing the biomarker studies closed and we were unable to find a replacement lab to run the samples. As a result, we are not able to make conclusive statements about the effect of cabozantinib on bone biomarkers. Of the 16 patients that were evaluable for determination of response by bone biomarkers, 13 had ≥ 40% decrease in serum Ctx, 9 had ≥ 40% decrease in serum Ntx, and 8 had ≥ 40% decrease in urinary Ntx. Values in Table 3 represent the maximum percent change in biomarker levels when compared to the level at screening. Percentage change in RECIST refers to maximum change target lesions as defined by RECIST version 1.1 (Table 3). These measurements, however, had low correlation with RECIST measurements (*r*^2^ of .05, .00, and .15, respectively).

**Table 3.** Changes in serum Ctx, serum Ntx, and urine Ntx.

% Change in serum Ctx	% Change in serum Ntx	% Change in urine Ntx	% Change in RECIST
−46.6	+9	na	−55.2
−48.3	−15	+400	+1.6
−29.7	+54	−61.1	−9.0
−72.4	−43.1	−59.4	−5.5
−92.3	−85.2	−84.9	−51.0
−84.4	−64.9	−84.8	+3.1
−64.7	−48.1	+44	−0.4
−65	−52.2	−69	−9.2
40.9	+19.5	186	na
−80.9	−51.9	−86.7	−12.3
−53.3	52.2	13.8	n/a
−81.8	−51.1	−66.7	−13.5
30	−13.1	+31.4	−2.2
−62.5	−42.2	−52.7	−37.3
−28.6	−44.7	+25	−5.7
66.7	n/a	n/a	−12.2

Abbreviations: Ctx, C-telopeptide; Ntx, N-telopeptide; RECIST, Response Evaluation Criteria in Solid Tumors.

## Conclusion

The primary objective of this phase II study was to test cabozantinib as a possible novel therapy for patients with bone metastasis derived from solid tumor malignancies. Indeed, 50% of the study population were progression-free by 3.5 months in this cohort of heavily pretreated patients with a variety of different cancers, and no SREs were observed during this study. In addition, we observed that serum Ctx and Ntx decreased as a result of cabozantinib administration, suggesting that cabozantinib was effective in reducing bone turnover. We were unable to find a correlation between the degree of change in Ctx or Ntx with tumor response as measured by RECIST. However, we were only able to collect complete sets of biomarkers on 16 patients, so this analysis was underpowered to detect a meaningful correlation.

Overall, cabozantinib was reasonably well tolerated in this cohort of patients. Fatigue and diarrhea were the most common adverse events. However, for most patients, these adverse events were manageable by a dose reduction to 40 mg per day.

Surprisingly, we also observed that cabozantinib had anti-tumor activity as determined by frequent and significant reductions in measurements of visceral metastasis, particularly in patients with sarcoma and radioiodine-refractory differentiated thyroid cancer. We also observed reduction in measurable disease burden in most of the patients who participated in this trial. This suggests that cabozantinib may have broad-spectrum anti-tumor activity across a large variety of cancer types. Importantly, we showed that cabozantinib has significant anti-tumor activity in patients with bone and soft-tissue sarcoma.

Additional studies are required to determine tumor-specific response rates and differential activity among the variety of malignancies shown to respond to cabozantinib therapy.

## Supplementary Material

oyac083_suppl_Supplementary_Material

## Data Availability

The data underlying this article will be shared on reasonable request to the corresponding author.
