# Supplementary material for: Phase II Study of Cabozantinib in Patients With Bone Metastasis
Source: Oncologist. 2022 May 7;27(7):600–6. doi: 10.1093/oncolo/oyac083 (PMC9256024; doi:10.1093/oncolo/oyac083)
Supplement: oyac083_suppl_Supplementary_Material [file oyac083_suppl_supplementary_material.pdf]

Phase II trial of cabozantinib (XL184) in patients with advanced solid (non-breast, non-prostate) malignancies and bony metastases

PROTOCOL NUMBER: 12-091

STUDY DRUG: Cabozantinib (XL184)

IND NUMBER: 115166

SPONSOR: Rebecca Heist, MD MPH

MEDICAL MONITOR: TBD

DATE: January 5, 2015

SYNOPSIS

TITLE

Phase II trial of cabozantinib (XL184) in patients with advanced solid malignancies and bony metastases

CLINICAL PHASE

Phase II

RATIONALE

Bone metastases are a common clinical problem in solid malignancies and cause significant symptomatic burden. Data from early clinical studies suggest that cabozantinib has a broad range of anti-tumor activity against multiple solid tumor types, as well as intriguing activity against bone metastases. We propose a phase II clinical trial to further explore the special effects cabozantinib may have on bony metastases from solid tumors.

OBJECTIVES

The objectives of this study are:

Primary Objective

1. To assess the effect of cabozantinib on bone biomarkers of osteoblast and osteoclast activity (urinary NTx, serum NTx, and serum CTx, among others).

Secondary Objectives:

1. To assess rate of SRE and time to SRE in patients treated with cabozantinib. (SRE defined as pathologic fracture, cord compression, radiation or surgery to bone, hypercalcemia).
2. To assess quality of life as measured by pain and analgesic scores and the FACT-G.
3. To assess overall tumor response rate by RECIST if patient has RECIST evaluable disease.
4. To correlate response with tumor genotyping and MET amplification, where available.
5. To assess the response to cabozantinib in bone metastatic disease as measured by bone scan or PET-CT scan.

STUDY DESIGN

A two-stage phase II design will be used, with an interim analysis and an early stopping rule for inactivity.

NUMBER OF SUBJECTS

Approximately 38 participants will participate in this study.

TARGET POPULATION

Cancer patients will be eligible for enrollment as defined by the inclusion and exclusion criteria as follows:

Inclusion Criteria
A patient must meet the following criteria to be eligible for the study:

1. The patient has a histologic or cytologic diagnosis of a solid tumor (not breast or prostate cancer) that is metastatic and is refractory to or progressed (or relapsed) following standard therapies, or has disease for which no standard therapy exists. Presence of metastatic bone lesion(s) is required.
2. The patient is ≥ 18 years old on the day of consent.
3. The patient has an Eastern Cooperative Oncology Group (ECOG) performance status of 0 or 1.
4. The patient has organ and marrow function and laboratory values as follows:
   a. Absolute neutrophil count (ANC) ≥ 1500/mm^{3}without colony stimulating factor support
   b. Platelets ≥ 100,000/mm^3
   c. Hemoglobin ≥ 9 g/dL
   d. Bilirubin ≤ 1.5 × the upper limit of normal (ULN). For patients with known Gilbert's disease, bilirubin ≤ 3.0 mg/dL
   e. Serum albumin ≥ 2.8 g/dl
   f. Serum creatinine ≤ 1.5 × ULN or creatinine clearance ≥ 50 mL/min. For creatinine clearance estimation, the Cockcroft and Gault equation should be used:
      Male: CrCl (mL/min) = (140 - age) × wt (kg) / (serum creatinine × 72)
      Female: Multiply above result by 0.85
   g. Alanine aminotransferase (ALT) and aspartate aminotransferase (AST) ≤ 2.5 × ULN if no liver involvement, or ≤ 5 × ULN with liver involvement
   h. Lipase < 1.5 x the upper limit of normal (except for patients with adenocarcinoma of the pancreas)
   i. Urine protein/creatinine ratio (UPCR) ≤ 1
   j. Serum phosphorus ≥ LLN
5. The patient is capable of understanding and complying with the protocol requirements and has signed the informed consent document.
6. Sexually active patients (men and women) must agree to use medically accepted barrier methods of contraception (eg, male or female condom) during the course of the study and for 4 months after the last dose of study drug(s), even if oral contraceptives are also used. All patients of reproductive potential must agree to use both a barrier method and a second method of birth control.
7. Women of childbearing potential must have a negative pregnancy test at screening. Women of childbearing potential include women who have experienced menarche and who have not undergone successful surgical sterilization (hysterectomy, bilateral tubal ligation, or bilateral oophorectomy) or are not postmenopausal. Post menopause is defined as:
   a. Amenorrhea ≥ 12 consecutive months. Note: women who have been amenorrheic for 12 or more months are still considered to be of childbearing potential if the amenorrhea is possibly due to prior chemotherapy, anti-estrogens, or ovarian suppression or any other reversible reason.

Exclusion Criteria
A patient who meets any of the following criteria is ineligible for the study:

1. Prior treatment with cabozantinib (XL184).
2. The patient has received cytotoxic chemotherapy (including investigational cytotoxic chemotherapy) or biologic agents (eg, cytokines or antibodies) within 3 weeks, or nitrosoureas/ mitomycin C within 6 weeks before the first dose of study treatment.
3. The patient has received radiation therapy:
   a. to the thoracic cavity or gastrointestinal tract within 3 months of the first dose of study treatment.
   b. to bone or brain metastasis within 14 days of the first dose of study treatment
   c. to any other site(s) within 28 days of the first dose of study treatment
4. The patient has received radionuclide treatment within 6 weeks of the first dose of study treatment.

5. The patient has received prior treatment with a small molecule kinase inhibitor or a hormonal therapy (including investigational kinase inhibitors or hormones) within 14 days or five half-lives of the compound or active metabolites, whichever is longer, before the first dose of study treatment.
6. The patient has received any other type of investigational agent within 28 days before the first dose of study treatment.
7. The patient has not recovered to baseline or CTCAE ≤ Grade 1 from toxicity due to all prior therapies except alopecia and other non-clinically significant AEs.
8. The patient has a primary brain tumor.
9. The patient has active brain metastases or epidural disease (Note: Patients with brain metastases previously treated with whole brain radiation or radiosurgery or patients with epidural disease previously treated with radiation or surgery who are asymptomatic and do not require steroid treatment for at least 2 weeks before starting study treatment are eligible. Neurosurgical resection of brain metastases or brain biopsy is permitted if completed at least 3 months before starting study treatment. Baseline brain scans are not required to confirm eligibility.)
10. The patient has prothrombin time (PT)/ International Normalized Ratio (INR) or partial thromboplastin time (PTT) test results at screening ≥ 1.3 × the laboratory ULN.
11. The patient requires concomitant treatment, in therapeutic doses, with anticoagulants such as warfarin or warfarin-related agents, heparin, thrombin or FXa inhibitors, or antiplatelet agents (eg, clopidogrel). Low dose aspirin (≤ 81 mg/day), low-dose warfarin (≤ 1 mg/day), and prophylactic low molecular weight heparin (LMWH) are permitted.
12. The patient has experienced any of the following within 3 months before the first dose of study treatment:
    a. clinically-significant hematemesis or lower gastrointestinal bleeding
    b. hemoptysis of ≥ 0.5 teaspoon (2.5 mL) of red blood
    c. any other signs indicative of pulmonary hemorrhage
13. The patient has radiographic evidence of cavitating pulmonary lesion(s) or tumor in contact with, invading or encasing major blood vessels
14. The patient has uncontrolled, significant intercurrent or recent illness including, but not limited to, the following conditions:
    a. Cardiovascular disorders including
        i. Congestive heart failure (CHF): New York Heart Association (NYHA) Class III (moderate) or Class IV (severe) at the time of screening
        ii. Uncontrolled hypertension defined as sustained BP > 140 mm Hg systolic, or > 90 mm Hg diastolic despite optimal antihypertensive treatment (BP must be controlled at screening)
        iii. Any of the following within 6 months before the first dose of study treatment:
            - unstable angina pectoris
            - clinically-significant cardiac arrhythmias
            - stroke (including TIA, or other ischemic event)
            - myocardial infarction
            - thromboembolic event requiring therapeutic anticoagulation
              (Note: patients with a venous filter (e.g. vena cava filter) are not eligible for this study)
    b. Gastrointestinal disorders particularly those associated with a high risk of perforation or fistula formation including:
        i. Any of the following at the time of screening
            - intra-abdominal tumor/metastases invading GI mucosa
            - active peptic ulcer disease,
            - inflammatory bowel disease (including ulcerative colitis and Crohn’s disease), diverticulitis, cholecystitis, symptomatic cholangitis or appendicitis

   ii. Any of the following within 6 months before the first dose of study treatment:
      (1) history of abdominal fistula
      (2) gastrointestinal perforation
      (3) bowel obstruction or gastric outlet obstruction
      (4) intra-abdominal abscess. Note: Complete resolution of an intra-abdominal abscess must be confirmed prior to initiating treatment with cabozantinib even if the abscess occurred more that 6 months ago.
   iii. GI surgery (particularly when associated with delayed or incomplete healing) within 28 days. Note: Complete healing following abdominal surgery must be confirmed prior to initiating treatment with XL184 even if surgery occurred more that 28 days ago.
   c. Other disorders associated with a high risk of fistula formation including PEG tube placement within 3 months before the first dose of study therapy or concurrent evidence of intraluminal tumor involving the trachea and esophagus.
   d. Other clinically significant disorders such as:
   i. active infection requiring systemic treatment
   ii. serious non-healing wound/ulcer/bone fracture
   iii. history of organ transplant
   iv. concurrent uncompensated hypothyroidism or thyroid dysfunction
   v. history of major surgery within 4 weeks or minor surgical procedures within 1 week before randomization

15. The patient is unable to swallow tablets
16. The patient has a corrected QT interval (QTcF) >500 ms within 28 days before randomization. If QTcF is $\geq$ 500 ms by Bazett formula, this must be confirmed by the Fridericia formula.
17. The patient is pregnant or breastfeeding.
18. The patient has a previously identified allergy or hypersensitivity to components of the study treatment formulation.
19. The patient is unable or unwilling to abide by the study protocol or cooperate fully with the investigator or designee.
20. The patient has had evidence within 2 years of the start of study treatment of another malignancy which required systemic treatment

ESTIMATED LENGTH OF SUBJECT PARTICIPATION

Subjects may continue to receive study treatment until they experience unacceptable drug-related toxicity or disease progression.

ESTIMATED STUDY DATES

4/2012 to 1/2016

INVESTIGATIONAL REGIMEN DOSE/ ROUTE/ DURATION

Cabozantinib is supplied as 20 and 60 mg tablets. Subjects will receive open-label cabozantinib at 60 mg daily. At the investigator’s discretion, the patient may at any time receive a reduced dose according to the dose reduction schedules in Section 3.3.

SAFETY ASSESSMENTS

Safety will be monitored on an ongoing basis. Laboratory testing (chemistry, hematology tests) will be performed every 2 weeks for the first 3 months followed by monthly assessments. Other safety evaluations including EKGs and urinalysis will be performed at regular intervals.

Adverse event seriousness, severity grade, and relationship to study treatment will be assessed by the investigator. Severity grade will be defined by the National Cancer Institute (NCI) Common Terminology Criteria for Adverse Events (CTCAE) version 4.0.

TUMOR ASSESSMENTS

Tumors will be assessed by restaging CT scans at a frequency of every 8 weeks.

BIOMARKER ASSESSMENTS

The primary endpoint of this trial is change in bone biomarkers, which will be assessed at baseline and during treatment. Bone biomarkers to be assessed are detailed in Section 5.5 and will be performed in real-time by the core clinical research laboratory at MGH.

STATISTICAL METHODS

The total sample size will be approximately 38 participants.

A two-stage phase II design will used, with an interim analysis and an early stopping rule for inactivity. The minimax design will be used. The first stage will include 19 evaluable participants. If at least 5 of these participants achieve a bony response to treatment, defined as ≥ 40% decrease in urinary Ntx, serum Ntx, or serum Ctx at week 8, enrollment will proceed with 10 additional participants. The underlying assumption is that the regimen will be of interest if the proportion of participants achieving the ≥ 40% decrease in urinary Ntx is ≥ 45%, and not of interest if the proportion achieving the endpoint is ≤ 20%. This design will guarantee an overall significance level of 0.05 with power of 90%. Of note 28 patients were required to achieve 19 evaluable patients for the first stage. Therefore the final accrual is being increased to 38, for an additional 10 pts after the first stage.

TABLE OF CONTENTS

1 BACKGROUND AND RATIONALE....13
1.1 Background: Cancer and Targeted Therapy....13
1.1.1 Signal Pathways....13
1.2 Cabozantinib Background....15
1.2.1 Spectrum of cabozantinib Activity....15
1.2.2 Cabozantinib Nonclinical Toxicology....17
1.2.3 Clinical Experience....17
1.2.3.1 Clinical Summary....17
1.2.3.2 Clinical Safety Profile....17
1.2.3.3 Clinical Pharmacokinetics....18
1.2.3.4 Clinical Activity....19
1.3 Rationale....22
1.3.1 Rationale for the Study....22
1.3.2 Rationale for Cabozantinib Dose Selection....22

2 STUDY OBJECTIVES AND DESIGN....23
2.1 Study Objectives....23
2.2 Study Design....23
2.2.1 Overview of Study Design....23
2.2.2 Study Treatment Period....23
2.2.3 Post-Treatment Period....23
2.3 Withdrawals....24

3 TREATMENTS....25
3.1 Composition, Formulation, and Storage....25
3.1.1 Investigational Treatment....25
3.2 Dose, Schedule and Route....25
3.3 Management of Adverse Events....26
3.3.1.1 General Guidelines for Non-Hematologic and Hematologic Adverse Events....26
3.3.1.2 Diarrhea, Nausea, Vomiting, Stomatitis, and Mucositis....29
3.3.1.3 Hepatobiliary Disorders....30
3.3.1.4 Pancreatic Conditions....32
3.3.1.5 Skin Disorders....33

3.3.2 Embolism and Thrombosis 35
3.3.2.1 Hypertension 35
3.3.2.2 Proteinuria 36
3.3.2.3 Hemorrhage 37
3.3.2.4 Rectal and Perirectal Abscess 38
3.3.2.5 Wound healing and Surgery 38
3.3.2.6 Endocrine Disorders 38
3.3.2.7 Gastrointestinal perforation and GI fistula 39
3.4 Concomitant Medications and Therapies 40
3.4.1 Anticancer Therapy 40
3.4.2 Other Medications 40
3.4.3 Potential Drug Interactions 41
3.5 Compliance 42
3.6 Study Drug Accountability 42

4 STUDY POPULATION 43
4.1 Inclusion Criteria 43
4.2 Exclusion Criteria 44
4.3 Registration Procedures 46

5 STUDY ASSESSMENTS AND PROCEDURES 48
5.1 Required Data 48
5.2 Pre-Treatment Period 50
5.2.1 Screening Assessments 50
5.3 Study Treatment Period 50
5.4 Post-Treatment Period 51
5.5 Electrocardiogram (ECG) Assessments 51
5.6 Vital Signs 52
5.7 Physical Examinations 52
5.8 Pain/Analgesic Assessment and QOL assessment 52
5.9 Laboratory Assessments 53
5.10 Tumor Assessment 54
5.10.1 Routine Tumor Assessment 54
5.10.2 Bone imaging 54

6 SAFETY 55
6.1 Adverse Events and Laboratory Abnormalities 55
6.1.1 Adverse Events 55
6.1.2 Serious Adverse Events 55
6.1.3 Serious Adverse Event Reporting 56
6.1.4 Regulatory Reporting 56

6.2 Other Safety Considerations 57
6.2.1 Laboratory Data 57
6.2.2 Pregnancy 57
6.2.3 Medication Errors/ Overdose 57
6.2.4 Follow-Up of Adverse Events 57

7 STATISTICAL CONSIDERATIONS 57
7.1 Analysis Population 57
7.1.1 Safety Population 57
7.2 Safety Analysis 57
7.2.1 Adverse Events 57
7.3 Sample Size 58

8 DATA QUALITY ASSURANCE 59

9 ETHICAL ASPECTS 59
9.1 Local Regulations 59
9.2 Informed Consent 59
9.3 Institutional Review Board/ Ethics Committee 60

10 CONDITIONS FOR MODIFYING THE PROTOCOL 60

11 CONDITIONS FOR TERMINATING THE STUDY 60

12 STUDY DOCUMENTATION AND RECORDKEEPING 60
12.1 Investigator's Files and Retention of Documents 60
12.2 Source Documents and Background Data 61
12.3 Audits and Inspections 61
12.4 Case Report Forms 61

13 MONITORING THE STUDY 62

14 CONFIDENTIALITY OF TRIAL DOCUMENTS AND SUBJECT RECORDS ... 62

15 PUBLICATION OF DATA AND PROTECTION OF TRADE SECRETS 62

16 REFERENCES 63

LIST OF TABLES

Table 1-1: Inhibition of Key Protein Kinases by XL184 Kinase 15
Table 1-2: Cabozantinib ED50 values in Tumor Efficacy Models 16
Table 1-3: Response Rate and Progression-Free Survival in XL184-201 20
Table 3-1 Dose Modification Levels 25
Table 3-2: Management of Non-Hematologic AEs 28
Table 3-3: Management of Hematologic AEs 29
Table 3-4: Management of ALT/AST Elevations 31
Table 3-5: Management of Bilirubin Elevations 32
Table 3-6: Management of Asymptomatic Lipase/Amylase Elevations 33
Table 3-7: Management of Symptomatic Pancreatitis 33
Table 3-8: Management of Hand Foot Skin Reactions 34
Table 3-9: Management of Hypertension Related to Cabozantinib 36
Table 3-10: Management of Treatment Emergent Proteinuria 37
Table 5-1: Required Data 48
Table 5-2: Bone Biomarkers 54

LIST OF APPENDICES

Appendix A: Performance Status Criteria 66
Appendix B: Cabozantinib Tablet Components and Composition 67

LIST OF ABBREVIATIONS

AE	adverse event
ALT	alanine aminotransferase
ANC	absolute neutrophil count
AST	aspartate aminotransferase
AUC	area under the plasma drug concentration time curve
C_{max}	maximum plasma concentration
cPR	confirmed partial response
CR	complete response
CRF	case report form
CTCAE	Common Terminology Criteria for Adverse Events
CYP	cytochrome P450
DVT	deep vein thrombosis
EC	ethics committee
ECG	electrocardiogram
ECOG	Eastern Cooperative Oncology Group
ED_{50}	dose required for 50% inhibition
ESC	Exelixis Safety Committee
FDA	Food and Drug Administration
FLT3	FMS-like tyrosine kinase 3
FSH	follicle-stimulating hormone
GB	glioblastoma
GCP	Good Clinical Practice
GI	gastrointestinal
GEJ	gastroesophageal junction
GnRH	gonadotropin-releasing hormone
HCC	hepatocellular carcinoma
HGF	hepatocyte growth factor
IC_{50}	concentration required for 50% inhibition
ICH	International Conference on Harmonisation
IME	important medical event
INR	International Normalized Ratio
IRB	Institutional Review Board
LHRH	luteinizing hormone-releasing hormone
MedDRA	Medical Dictionary for Regulatory Activities
MTC	medullary thyroid cancer
MTD	maximum tolerated dose
NCI	National Cancer Institute

LIST OF ABBREVIATIONS (continued)

NSCLC	non-small-cell lung cancer
PD	progressive disease
PE	pulmonary embolism
PFS	progression-free survival
PFS6	progression-free survival at 6 months
PI	principal investigator
PIB	powder-in-bottle
PK	pharmacokinetic
PO	oral
PPE	palmar-plantar erythrodysesthesia
PR	partial response
PT	prothrombin time
PTT	partial thromboplastin time
qd	once daily
QTc	corrected QT
RPLS	reversible posterior leukoencephalopathy syndrome
RTK	receptor tyrosine kinase
SAE	serious adverse event
SD	stable disease
SCLC	small-cell lung cancer
$T_{1/2, z}$	terminal-phase half-life
ULN	upper limit of normal
VEGF(R)	vascular endothelial growth factor (receptor)

1 BACKGROUND AND RATIONALE

1.1 Background: Cancer and Targeted Therapy

Cancer is a worldwide clinical problem. Conventional approaches to treating cancer include surgery, radiotherapy, and cytotoxic chemotherapy as single modalities or as combined therapies. Recently, targeted therapies including antibodies and small molecule inhibitors have also demonstrated clinical benefit.

1.1.1 Signal Pathways

The MET receptor tyrosine kinase (RTK) (receptor for hepatocyte growth factor [HGF]) has been implicated as a mediator in many important aspects of tumor pathobiology, including tumor survival, growth, angiogenesis, invasion, and dissemination (Sattler et al. 2004; Jiang et al. 2005), and several MET RTKs have been reported to show activity in cell lines and animal models (Sattler et al. 2004). Recently, inhibitors of MET including XL880 and ARQ 197 have shown signs of antitumor activity in Phase 1 studies (Eder et al. 2007; Garcia et al. 2007; Ross et al. 2007). The vascular endothelial growth factor receptor 2 (VEGFR2 [KDR]) is a central mediator of tumor angiogenesis, and several small molecule and protein therapeutics targeting this receptor are currently in clinical development. Recently, bevacizumab (Avastin®), a monoclonal antibody directed against VEGF, has been shown to improve overall survival when combined with chemotherapy in patients with metastatic colorectal cancer (Hurwitz et al. 2004) and in lung cancer (Sandler et al. 2005). In addition to their individual roles in tumor pathobiology, nonclinical data suggest that Met and VEGFR2 play synergistic roles in promoting tumor angiogenesis and subsequent dissemination (Bottaro and Liotta 2003).

Compounds that simultaneously inhibit VEGF and MET RTKs may be more effective anticancer agents than agents that target each of these receptors individually (Pennacchietti et al. 2003). The investigational drug in this study, cabozantinib, is a potent RTK inhibitor that targets primarily MET and VEGFR2 RTKs. Cabozantinib has activity against other RTKs that have been implicated in tumor pathobiology, including KIT, FMS-like tyrosine kinase 3 (FLT3), and Tie-2. In addition, cabozantinib is known to inhibit RET, a RTK known to be causative for malignancy in the setting of hereditary medullary thyroid cancer (MTC).

1.1.2. Bone Metastases in Solid Malignancies

Bone is one of the most common sites of metastasis in solid malignancies, and a major cause of morbidity for patients suffering skeletal-related events (SREs) such as pain, hypercalcemia, pathologic fractures, or cord compression (Mundy 1997). Bone metastases can be osteolytic or osteoblastic, or commonly a mixture of both types (Coleman 1997). A “vicious cycle” has been described where tumor cells secrete factors that stimulate osteoclast-mediated bone destruction and osteoblast-mediated abnormal bone formation, and the dysregulated bone turnover releases growth factors from the bone microenvironment that stimulate tumor cell growth (Mundy et al. 2002; Guise et al. 2006). Although bisphosphonates can decrease SREs and time to SRE, the rates of SREs are still high in the non-breast, non-prostate, solid tumor population (47% in placebo arm, 38% in zoledronic acid arm in Phase III trial) (Rosen et al. 2003), and complications of bone metastases remain a major cause of pain and decreased quality of life for patients.

Interestingly, in addition to anti-tumor response at sites of measurable disease, cabozantinib has also demonstrated the ability to significantly reduce bone scan radiotracer uptake in patients with bone metastases. The mechanism for this activity has not yet been fully elucidated, nor is it fully understood if cabozantinib is having direct anti-tumor effects or is altering bone metabolism. Preclinical models described more fully in Section 1.2.1.1 below suggest cabozantinib has activity against both osteoblastic and osteoclastic activity in xenograft models of tumor in bone. The two main kinase targets of cabozantinib, MET and VEGFR, may play a role in formation and progression of bony metastases.

1.1.3. Biomarkers of response in bone

Despite the importance of bone metastases for both morbidity and mortality from advanced solid tumors, there is no clear consensus on how best to measure response to cancer therapeutics in bone. Bone metastases are considered "unmeasurable" by standard RECIST criteria, and indeed there is no consensus on the best imaging modality for evaluating bone metastases (Hamaoka et al. 2004). While bone scan has historically been used, it is a measure of osteoblastic activity only, and may not be optimal for lytic lesions, where FDG-PET may be more sensitive. Regardless of imaging modality, monitoring response on scans can be challenging in light of the "flare phenomenon" which can happen in bony lesions undergoing response to treatment and the delayed time frame in which a true response is observed (Hamaoka et al. 2004).

Biomarkers of bone activity may be a useful surrogate to measure response in bone metastases, and many clinical trials have used bone biomarkers as surrogate endpoints (Brown et al. 2010). Markers of bone metabolic turnover such as urinary N-telopeptide (Ntx), among others, have been measured in patients with metastatic bone disease and have been correlated with clinical outcomes, both with and without concurrent bisphosphonate therapy (Coleman et al. 2005; Brown et al. 2005; Demers et al. 2000; Lipton et al. 2008; Cook et al. 2006). Patients with metastatic bone disease in both the treatment and placebo arms in Phase III trials of zoledronic acid were found to have higher risk of SREs and death when urinary Ntx markers were high (Coleman et al. 2005; Brown et al. 2005). In addition, normalization of urinary Ntx after treatment with zoledronic acid correlated with improved survival and SRE-free survival (Lipton et al. 2008). Which biomarker is most relevant remains unclear; on multivariate analysis serum BAP remained significantly associated with overall survival among men with prostate cancer and bone metastases, but not urinary Ntx (Cook et al. 2006). Other biomarkers of bone activity, such as serum C-telopeptide (Ctx) may also be important in assessing response in bone (Smith et al. 2009), and most studies test a panel of bone biomarkers encompassing most osteoblastic and osteolytic activity. The ability to measure dynamic real-time changes in bone biomarker levels may ultimately prove them more clinically useful measures of treatment response than imaging modalities.

1.1.4. Cabozantinib and bone metastases

We propose a phase II clinical trial to further explore the special effects cabozantinib may have on bony metastases from solid tumors. In addition to the clinical experience with cabozantinib which is summarized in section 1.2.3 below, there has been intriguing activity seen against bone metastases with cabozantinib. In XL184-203, a randomized discontinuation study across a broad range of tumor types, the observed overall response rate was very promising, with 39 PRs and 1 CR seen among 490 evaluable patients. In addition to anti-tumor activity, response in bone with

concomitant decrease in serum biomarkers such as CTx was seen. Among patients with metastatic prostate cancer and bony metastases, cabozantinib led to partial or complete resolution of lesions on bone scan in 76% (82/108) of evaluable patients, stable disease in 21% (23/108), and progressive disease in 3% (3/108). 68% (56/83) of those with bone pain at presentation reported improvement on study. Among both patients who were treated with bisphosphonates and those who were bisphosphonate-naïve, decreases in bone biomarkers such as alkaline phosphatase and CTx were seen. Similar responses on bone scan were also seen in melanoma and breast cancer. Given the broad range of antitumor activity across many solid tumor types of cabozantinib (see clinical activity section 1.2.3.4 below) as well as the large burden of disease of bony metastases from solid malignancies, we propose to investigate the activity of cabozantinib in solid malignancies (non-breast, non-prostate, as there will be separate studies for these populations) with bony metastases.

Our primary endpoint will be change in levels of bone biomarkers in response to treatment with cabozantinib. In our secondary endpoints, we will seek to elucidate the effect of cabozantinib on major indices of quality of life, by assessing time to SRE and rate of SRE, and by using specific pain and analgesic scores and QOL questionnaires. In addition, we will seek to correlate bone biomarker response with response on imaging studies, both bone-specific response on bone scan or PET, and overall tumor response by RECIST. We will incorporate bone scan and PET response in the second stage of the study, if the study meets criteria to proceed to second stage. Finally, we will correlate response with tumor genetic markers including MET amplification.

1.2 Cabozantinib Background

A summary of cabozantinib clinical and nonclinical experience is contained in the Investigator's Brochure supplied by Exelixis. The Investigator's Brochure should be reviewed in conjunction with this study protocol.

1.2.1 Spectrum of cabozantinib Activity

Cabozantinib exhibits potent inhibitory activity against several receptor tyrosine kinases that are known to influence tumor growth, metastasis, and angiogenesis. The primary targets of cabozantinib are MET and VEGFR2/KDR, with IC_{50} (concentration associated with 50% inhibition) values of 1.8 and 0.035 nM, respectively. The in vitro target inhibition profile of cabozantinib is shown in Table 1-1.

Table 1-1: Inhibition of Key Protein Kinases by cabozantinib

Kinase	IC_{50} ± SEM (nM)
MET	1.8 ± 0.2
RET	9.8 ± 2.3
VEGFR2/KDR	0.035 ± 0.007
VEGFR1/FLT-1	12.2 ± 0.7
VEGFR3/FLT-4	6.0 ± 0.6
AXL	7
TIE-2	14.3 ± 2.8
KIT	4.6 ± 0.5
FLT-3	14.4 ± 0.8

Data from pharmacodynamic experiments have shown that cabozantinib inhibits MET and VEGFR2 in vivo. Oral administration of cabozantinib resulted in blockade of MET phosphorylation in human lung tumor xenografts in nude mice and blockade of VEGFR2 phosphorylation in mouse lung tissue. The duration of action for cabozantinib was sustained, with > 50% inhibition observed 10-24 hours post-dose at a dose level of 100 mg/kg for all targets studied.

Treatment with cabozantinib results in anti-angiogenic effects in xenograft tumors, with disruption of the vasculature beginning within 24 hours after administration. These effects translate into significant tumor growth inhibition or tumor regression after cabozantinib treatment in multiple tumor models including MTC (thyroid), breast cancer, lung carcinoma, and GB (brain) (Table 1-2).

Table 1-2:Cabozantinib ED_{50} Values in Tumor Efficacy Models

Tumor Cell Line	Species	Tissue of Origin	ED_{50} (mg/kg/day)	Treatment Duration
C6	Rat	Brain	< 1	qd × 12
MDA-MB-231	Human	Breast	2	qd × 14
H441	Human	Lung	3	qd × 14
TT	Human	Thyroid	11	qd × 21

ED_{50}, dose associated with 50% tumor growth inhibition; qd, once daily.

1.2.1.1. Activity of cabozantinib in a Preclinical Bone Metastasis Model

The MET and VEGF signaling pathways appear to play important roles in osteoblast and osteoclast function. Strong immunohistochemistry (IHC) staining of MET has been observed in both cell types in developing bone (Leonardi et al. 2010). HGF and MET are expressed by osteoblasts and osteoclasts in vitro and mediate cellular responses such as proliferation, migration, and expression of alkaline phosphatase (ALP) (Inaba et al. 1993, Grano et al. 1996). Osteoblasts and osteoclasts also express VEGF and its receptors, and VEGF signaling in these cells is involved in potential autocrine and/or paracrine feedback mechanisms regulating cell migration, differentiation, and survival (Zelzer and Olsen 2005, Street and Lenehan 2009). Secretion of HGF by osteoblasts has been proposed as a key factor in osteoblast/osteoclast coupling (Grano et al. 1996) and in the development of bone metastases by tumor cells that express MET (Ono et al. 2006). Prominent expression of MET has been observed in primary and metastatic prostate carcinomas (Pisters et al. 1995, Humphrey et al. 1995) with evidence for higher levels of expression in bone metastases compared to lymph node metastases or primary tumors (Knudsen et al. 2002, Zhang et al. 2010).

The human prostate cancer model ARCaP-M, which expresses both MET and the VEGF co-receptor NP-1 (Zhang et al 2010), was used in a prostate tumor xenograft study in bone. ARCaP-M cells were injected into the tibiae of nude mice on Day 1, and on Day 31 animals with established bone lesions were randomized to receive cabozantinib or vehicle qd for 7 weeks of treatment. Tibiae from vehicle-treated animals exhibited both osteoblastic and osteolytic lesions, whereas tibiae from cabozantinib-treated animals appeared mostly normal. Thus, cabozantinib treatment blocked both osteoblastic and osteolytic progression of ARCaP-M xenograft tumors in bone.

More extensive summaries of cabozantinib pharmacology are contained in the Investigator's Brochure supplied by Exelixis. This document should be reviewed in conjunction with this study protocol.

1.2.2 Cabozantinib Nonclinical Toxicology

In nonclinical toxicity studies in rodents and non-rodents, histopathological changes associated with cabozantinib administration were observed in gastrointestinal (GI) tract, bone marrow, lymphoid tissues, kidney, adrenal and reproductive tract tissues. Histopathological changes present in bone and pancreas were considered secondary to cabozantinib administration. cabozantinib was negative in in vitro bacterial, in vitro mammalian cell, and in vivo mammalian genotoxicity bioassays. In reproductive toxicity studies, cabozantinib was embryotoxic in rats, produced fetal soft tissue changes in rabbits, and decreased fertility in male and female rats.

Safety pharmacology studies of cabozantinib administration did not demonstrate adverse effects on neurobehavioral or respiratory-system function in rats; furthermore, no significant changes in electrocardiographic parameters (including corrected QT [QTc] interval) were observed in telemeterized dogs.

Additional toxicology information may be found in the Investigator's Brochure.

1.2.3 Clinical Experience

1.2.3.1 Clinical Summary

As of 04 May 2011, 1003 subjects have been enrolled in open-label clinical studies of cabozantinib, and 330 subjects have been enrolled in a placebo-controlled blinded Phase 3 study. Clinical data are available from nine studies of cabozantinib including four Phase 1 studies, one Phase 1b/2 study, three Phase 2 studies, and one Phase 3 study. Details of all studies may be found in the Investigator's Brochure.

1.2.3.2 Clinical Safety Profile

1.2.3.2.1 Adverse Events

As of 01 March 2011, AE data are available for 913 subjects who have been dosed with cabozantinib in open-label clinical studies (806 subjects in single-agent cabozantinib studies and 107 subjects in combination studies of cabozantinib with erlotinib, rosiglitazone, or TMZ ± radiation).

Based on data available as of 01 March 2011, the most frequently (> 20%) observed AEs, regardless of causality, were fatigue, diarrhea, nausea, decreased appetite, constipation, PPE syndrome, vomiting, dysphonia, and hypertension. The safety profile of single agent cabozantinib is consistent across tumor types. The most common AEs reported at severity of Grade 3 and above include fatigue, PPE, diarrhea, blood lipase increased, hypertension, hypophosphatemia, abdominal pain, dehydration, PE and DVT.

Effects that may be related to inhibition of VEGF, including hypertension, thromboembolic events, GI perforation and hemorrhage, wound dehiscence, and proteinuria, have been observed in clinical studies with cabozantinib.

1.2.3.2.2 Serious Adverse Events

As of 04 May 2011, of the 1003 subjects enrolled in open-label clinical trials with cabozantinib (either as a single-agent or in combination with other therapies), 473 subjects (47%) experienced one or more SAEs, and 199 subjects experienced one or more SAEs that was assessed as related to treatment with cabozantinib. The majority of SAEs were attributed to the underlying cancer.

Across all open-label studies, the most common SAEs, regardless of causality were PE, dehydration, vomiting, DVT, pneumonia, diarrhea, nausea, convulsion, mental status changes, and abdominal pain.

As of 04 May 2011, across all open-label studies (single-agent cabozantinib and cabozantinib in combination with other therapies), 111 fatal SAEs were reported. The majority of these SAEs were attributed to disease progression. Twenty-eight of the fatal SAEs were attributed to causes other than disease progression (see IB, Table 5-7 and Table 5-8). Of the 28 fatal SAEs, 15 were assessed as related to study treatment (cabozantinib as a single agent or in combination with other therapies). Short narratives are provided for all deaths, regardless of causality, that occurred on study and are not attributed to disease progression (see IB).

Detailed information regarding the safety profile of cabozantinib from all studies may be found in the Investigator's Brochure.

1.2.3.3 Clinical Pharmacokinetics

Details can be found in the Investigator Brochure.

The following pharmacokinetic (PK) data regarding was available after finalization of the current IB:

Comparative Bioavailability Study of Cabozantinib Tablet and Capsule Formulations in Healthy Adult Subjects (Study XL184-005)

Study XL184-005 is a Phase 1, open-label, randomized, single-dose, two-treatment, two-way crossover comparative bioavailability study of cabozantinib tablet and capsule formulations in healthy volunteers. Subjects received single oral doses of the assigned treatment of Test (100 mg cabozantinib, dosed as one 100-mg tablet) or Reference (100 mg cabozantinib, dosed as two 50-mg capsules), according to a randomization scheme. Each dosing was administered under fasting conditions, and blood samples were collected up to 504 hours post-dose for each subject after each treatment to assess plasma cabozantinib PK.

Based on the preliminary PK data from 23 subjects who completed both treatments, after a single oral dose of cabozantinib at 100 mg, the terminal-phase half-life ($t_{1/2, z}$) of cabozantinib appeared to be similar for both tablet and capsule formulations, with approximately mean values of 110 hours. The median time to the maximum plasma concentration (t_{max}) was 4 hours for the tablet formulation and 5 hours for the capsule formulation. High inter-subject variability for the maximum plasma concentration (C_{max}) and the area under the plasma drug concentration time curve (AUC) values were observed for both formulations (coefficient of variation [CV]% C_{max}: 51% for the tablet formulation, 61% for the capsule formulation; CV% for the AUC from time zero to the last quantifiable time point or to infinity [$AUC_{0\text{-}last}$ or $AUC_{0\text{-}inf}$]: 40-43% for the

tablet formulation, 43% for the capsule formulation.) The geometric mean C_{max} of the tablet formulation was approximately 49% higher than the value observed for the capsule formulation. The geometric mean $AUC_{0\text{-}last}$ and $AUC_{0\text{-}inf}$ values for the tablet formulation were also higher (15% and 19%, respectively) than those observed for the capsule formulation. However, due to the high within-formulation variability observed, no statistical difference in exposure between the two formulations was apparent.

Effect of Food on the Bioavailability of Cabozantinib in Healthy Adult Subjects (Study XL184-004)

Study XL184-004 is a Phase 1, open-label, randomized, single-dose, two-treatment, two-way crossover study to assess the effect of food on the bioavailability of cabozantinib in healthy adult subjects. According to a randomization scheme, 56 subjects received single oral doses of the assigned treatment of Test (175 mg cabozantinib, dosed as one 100-mg capsule and three 25-mg capsules 30 minutes after administration of a high-fat breakfast) or Reference (175 mg cabozantinib, dosed as one 100-mg capsule and three 25-mg capsules under fasting conditions). Blood samples were collected up to 504 hours post-dose for each subject after each treatment to assess plasma cabozantinib pharmacokinetics.

Based on the preliminary PK data from 46 subjects who completed both treatments, a high-fat meal did not appear to alter the terminal $t_{1/2,\,z}$ of cabozantinib [mean $t_{1/2,\,z}$: 131 hours (fed) vs 128 hours (fasted)]. The high-fat meal significantly increased the median t_{max} to 6 hours from 4 hours (fasted). The high-fat meal also significantly increased both the cabozantinib C_{max} and AUC values by 39% and 56%, respectively. The geometric mean ratio of C_{max} fed/fasted was 1.39 (90% CI: 1.16-1.67), and the geometric mean ratio of $AUC_{0\text{-}last}$ fed/fasted was 1.56 (90% CI: 1.34-1.80). Based on this result, cabozantinib should be taken on an empty stomach (fasting is required 2 hours before and 1 hour after each cabozantinib dose).

1.2.3.4 Clinical Activity

Preliminary clinical activity data are available for Studies XL184-001, XL184-201, XL184-202, and XL184-203.

Study XL184-001 was designed primarily as a safety and PK study to determine the maximum tolerated dose and included an exploratory endpoint of antitumor activity. The dose escalation phase was in subjects with advanced cancer, and a dose expansion phase was conducted primarily in subjects with MTC. A total of 85 subjects, including 37 subjects with MTC, were enrolled in the XL184-001 study.

Among the 35 subjects with MTC and measurable disease, cPR was achieved in 10 subjects (29%; 95% CI, 15-45%), and a 30% or greater decrease in the sum of tumor measurements compared with baseline measurements was achieved in 17 subjects (49%). Five of the 10 responders had a PR at the first radiologic assessment. Onset of tumor response in the MTC population was reported as early as Day 21 and as late as Day 365, and median time to response was 49.5 days. The median duration of response has not been reached (range, 3.9 to 35+ months). Overall, SD of at least 6 months duration (range, 6.4 to 31.1 months) was observed in 15/37 (41%) of MTC subjects, and response duration of at least 6 months has been reported in 7 subjects. Activity was independent of both RET mutation status and prior treatment with

tyrosine kinase inhibitors, including those known to inhibit RET (eg, vandetanib) (Kurzrock et al. 2011). In an update from 06 July 2011, 8 subjects (7 with MTC and 1 with papillary DTC) remain on study (time on study is 36 to 56 months), including one subject with a duration of response reported to be up to 48+ months (Exelixis, Inc. 2011).

Study XL184-201 was designed to evaluate the safety and tolerability and antitumor activity of cabozantinib in subjects with progressive or recurrent GBM. Clinical efficacy data are summarized in Table 1-3 for 46 subjects who received cabozantinib at a starting dose of 175 mg (Group A) and 59 subjects who received cabozantinib at a starting dose of 125 mg qd (Group B). In both of these cohorts, radiographic response was evaluated by an IRF review of MRI scans per modified Macdonald criteria (Macdonald et al. 1990). An additional cohort with a starting dose of 125 mg qd (Group C) was also enrolled, but clinical efficacy data have not yet been analyzed.

Table 1-3: Response Rate and Progression-Free Survival in XL184-201 (N = 105)

	Prior Anti-Angiogenic Treatment			
	Naïve		Pretreated	
Group	A (n = 34)	B (n = 37)	A (n = 12)	B (n = 22)
Dose	175 mg	125 mg	175 mg	125 mg
ORR, n (%)[a]	7 (21)	11 (30)	1 (8)[b]	0
Median duration of response, months (range)[a]	2.9 (1.9-12.8)	5.1 (0.9+ - 6.7+)	NE	NE
Median progression-free survival, weeks[c]	15.9	16.0	14.3	7.9
Progression-free survival at 6 months, %[c]	10	25	38	0

RF, independent radiology facility; NE, not estimable; ORR, objective response rate.
Per IRF
Duration of response = 12.3 months (subject previously treated with vandetanib)
Per investigator

Study XL184-202 was designed to evaluate the safety and tolerability of cabozantinib and erlotinib administered in combination and to estimate the antitumor activity of cabozantinib with and without erlotinib in subjects with NSCLC.

Sixty-four subjects were enrolled in the Phase 1 dose-escalation portion of the study examining the combination of cabozantinib and erlotinib in NSCLC subjects. All but two subjects had been previously treated with and progressed on erlotinib therapy. Nine subjects (14%) had a ≥ 30% decrease in the sum of tumor measurements compared with baseline measurements. A cPR was observed in 5 subjects (8%). In addition, 24 subjects (37%) had SD/PR ≥ 4 months (range, 4.6-23+ months).

Twenty-eight subjects were enrolled in the Phase 2 portion of the study, in which subjects who had received clinical benefit from erlotinib and subsequently experienced PD receive single-agent cabozantinib or cabozantinib in combination with erlotinib. Two subjects (7%) had a ≥ 30% decrease in the sum of tumor measurements compared with baseline measurements (one subject who received treatment with single-agent cabozantinib and one subject who received treatment with cabozantinib in combination with erlotinib). A cPR was observed in the subject who was treated with single-agent cabozantinib.

In Study XL184-203, an ongoing Phase 2 randomized discontinuation study of cabozantinib in subjects with advanced solid tumors, a total of 531 subjects have been enrolled. An analysis of data from the Lead-In Stage for 490 subjects showed clinical activity in multiple tumor types including regression of soft-tissue tumor disease, bone scan resolution in subjects with bone metastasis, and other signs of clinical benefit (Gordon et al. 2011; Hussain et al. 2011; Bukanovich et al 2011). Randomization was suspended in the CRPC and ovarian cancer cohorts based on observed high rates of clinical activity, and randomized subjects were unblinded. Non-randomized expansion (NRE) cohorts have been initiated for these tumor types.

The overall disease control rates (partial response [PR] + SD) at Week 12 were 73% in the HCC cohort, 53% in the ovarian cancer cohort, 68% in the prostate cancer cohort, 47% in the melanoma cohort, 45% in the breast cancer cohort, and 40% in the NSCLC cohort.

1.2.3.5. Activity in Bone

In Study XL184-203, among 108 CRPC subjects evaluable for post-baseline bone scan changes, best assessments of bone scan were partial or complete resolution in 82 subjects (76%), stable disease in 23 subjects (21%), and PD in three subjects (3%). Based on a retrospective survey completed by investigators, the majority of the subjects reported reduced bone pain and reduced reliance upon narcotic pain medication. There were 83 subjects with bone metastases and bone pain at baseline who had at least one post-baseline assessment of pain status. Of these, 56 subjects (68%) had pain improvement at either Week 6 or 12. There were 71 subjects who required narcotic analgesic medication at baseline for control of bone pain. Among the 67 of these subjects who were evaluable for post-baseline pain improvement, 47 (70%) had pain improvement at Week 6 or Week 12, and among the 55 subjects who were evaluable for post-baseline changes in consumption of narcotics, 31 (56%) were able to decrease or discontinue narcotic medication.

A positive correlation between bone scan resolution and improvement in clinical symptoms of disease was observed. Subjects with bone scan resolution (either complete or partial) were more likely to be free of disease progression at 6 months (61% vs. 35%), experience pain relief (83% vs. 43%), reduce or eliminate their need for narcotic analgesics (68% vs. 33%), achieve tumor regression (78% vs. 58%), and experience substantial declines in markers of bone turnover (60% vs. 43%), as compared to those who did not achieve bone scan resolution (stable or progressing bone scan).

Reductions of t-ALP and CTx were observed. Of 28 subjects with bone metastases who had t-ALP levels at least 2 × ULN, and at least 12 weeks of follow-up, the majority had decreases in t-ALP. In addition, of 118 subjects with bone metastases and plasma CTx data, the majority also showed a decrease in CTx at Week 6 or Week= 12. Reductions in either CTx or t-ALP occurred regardless of prior bisphosphonate treatment.

1.3 Rationale

1.3.1 Rationale for the Study

Bone metastases are a common clinical problem in solid malignancies and cause significant symptomatic burden. Data from early clinical studies suggest that cabozantinib has a broad range of anti-tumor activity against multiple solid tumor types, as well as intriguing activity against bone metastases. We propose a phase II clinical trial to further explore the special effects cabozantinib may have on bony metastases from solid tumors. See sections 1.1.2 to 1.1.4 for further details of rationale.

1.3.2 Rationale for Cabozantinib Dose Selection

Cabozantinib at a starting dose of 100 mg qd has been studied in 171 CRPC subjects enrolled to a Phase 2 randomized discontinuation (RDT) study. Despite relatively high rates of cabozantinib dose reductions to the next lowest dose of 60 mg qd within the first 12 weeks of therapy (51%), this starting dose resulted in high rates of pain relief, bone scan improvement and overall disease control.

Preliminary data from a separate and ongoing dose-ranging study looking at lower doses of cabozantinib in CRPC coupled with results from a retrospective review of the Phase 2 RDT study indicate that lower doses below 100 mg qd are likely to retain efficacy while improving upon tolerability:

> <u>Preliminary results from an ongoing dose-ranging study</u>: To date, 9 subjects with metastatic CRPC enrolled to the first cohort (starting dose of 40 mg qd) are evaluable for bone scan response. All 9 subjects exhibit evidence of response on bone scan including two complete responses. Although most subjects did not have pain at baseline, one subject reported pain at baseline which resolved by Week 6. No dose reductions or interruptions have been reported to date, although one subject discontinued study treatment for fatigue that was present at baseline and another subject discontinued because of a pathological fracture. This provides preliminary evidence that lower doses are pharmacologically active in a patient population with advanced CRPC.
>
> <u>Retrospective review of Phase 2 RDT trial:</u> While the overall rate of dose reduction from 100 mg to 60 mg was 51%, only 14% required an additional reduction in dose from 60 mg to the next lowest dose of 40 mg, which is consistent with an overall improvement in tolerability profile at the 60 mg dose level. The majority (69%) of subjects with pain at baseline who experienced early dose reduction (before Week 6) to 60 mg went on to report pain improvement at Week 6. Moreover, 80% of these subjects remained progression-free and continued to report pain relief at the Week 12 time point. Thus the dose of 60 mg qd appears to offer improved tolerability while maintaining efficacy in a patient population with advanced CRPC and cancer-related pain at baseline.

Based on the above data, the cabozantinib dose and regimen adopted for this study is a starting dose of 60 mg qd. The goal of this regimen is to improve the overall tolerability of cabozantinib while prolonging survival in this patient population.

2 STUDY OBJECTIVES AND DESIGN

2.1 Study Objectives

The objectives of this study are as follows:

Primary Objective

1. To assess the effect of cabozantinib on bone biomarkers of osteoblast and osteoclast activity (urinary NTx, serum NTx, serum CTx, among others).

Secondary Objectives:

2. To assess rate of SRE and time to SRE in patients treated with cabozantinib. (SRE defined as pathologic fracture, cord compression, radiation or surgery to bone, hypercalcemia.
3. To assess quality of life as measured by pain and analgesic scores and the FACT-G.
4. To assess overall tumor response rate by RECIST if patient has RECIST evaluable disease.
5. To correlate response with tumor genotyping and MET amplification, where available.
6. To assess the response to cabozantinib in bone metastatic disease as measured by bone scan or PET-CT scan.

2.2 Study Design

2.2.1 Overview of Study Design

This is an open label Phase II study. A two-stage design will be employed, with early stopping rules for lack of activity and the ability to explore specific patient subgroups if promising activity is observed.

2.2.2 Study Treatment Period

The Study Treatment Period will consist of continued treatment during which time participants will receive cabozantinib until either disease progression or the occurrence of unacceptable drug-related toxicity. Participants will be assessed for safety on an ongoing basis. Laboratory testing (chemistry, hematology tests) will be performed every 2 weeks for the first 3 months followed by monthly assessments. Other safety evaluations including EKGs and urinalysis will be performed at regular intervals. Adverse event seriousness, severity grade, and relationship to study treatment will be assessed by the investigator. Severity grade will be defined by the National Cancer Institute (NCI) Common Terminology Criteria for Adverse Events (CTCAE) version 4.0.

Participants' tumors will be objectively assessed by restaging CT scans at a frequency of every two cycles (one cycle = 28 days). The purpose of these assessments is to allow the investigator to determine if the participant is benefiting from cabozantinib.

2.2.3 Post-Treatment Period

Participants will return to the study site to complete end-of-study assessments between 30 to 37 days after the last dose of cabozantinib.

Treatment Assignment

It is the responsibility of the investigator to assign a participant number prior to treating each participant with cabozantinib.

2.3 Withdrawals

Participants may discontinue study treatment or withdraw their consent to participate in the study at any time without prejudice. The investigator may withdraw a participant from study treatment or from the study if, in his or her clinical judgment, it is in the best interest of the participant or if the participant cannot comply with the protocol.

In addition, any of the following conditions require withdrawal of the participant from study treatment:

- An AE or intercurrent illness that in the opinion of the investigator warrants the participant's withdrawal from treatment
- Necessity for treatment with other investigational drug or other anticancer medications prohibited by protocol
- Noncompliance with the protocol schedule
- Participation in another clinical study using anticancer agent(s)
- Request by regulatory agencies for termination of treatment of an individual participant or all participants under this protocol
- Sexually active participants who refuse to use medically accepted barrier methods of contraception (eg, male condom, female condom) during the course of the study and for 3 months following discontinuation of study treatment
- Women who become pregnant or are breast feeding
- Inability to tolerate a dose of 20 mg of cabozantinib
- Cabozantinib treatment delays > 4 weeks unless the participant was unequivocally benefitting from cabozantinib treatment
- Development of tumors abutting, encasing, or invading a major blood vessel or development of cavitation of pulmonary tumor during study treatment
- Progressive disease (PD) as determined by the investigator.

The reason for study treatment discontinuation will be documented. For participants who discontinue or are withdrawn from study treatment, every effort must be made to undertake protocol-specified follow-up procedures and end-of-treatment assessments, if possible, unless consent to participate in the study is also withdrawn.

If a participant fails to return for the protocol-defined visits, an effort must be made to determine the reason. If the participant cannot be reached by telephone, at the minimum a registered letter should be sent to the participant (or the participant's legal guardian) requesting contact with the clinic.

If a participant is discontinued from study treatment because of an AE considered to be related to study treatment and the event is ongoing 30 days after the last dose of study treatment, the event must be followed until resolution or determination by the investigator that the event has become stable or irreversible.

If a participant withdraws consent to participate in the study, the reason for withdrawal will be documented, no further study procedures or assessments will be performed, and no further study data will be collected for this participant, other than the determination of survival status from public records such as government vital statistics or obituaries.

3 TREATMENTS

3.1 Composition, Formulation, and Storage

At study sites, all study medication will be stored as described in the pharmacy manual and inventoried in accordance with applicable state and federal regulations.

3.1.1 Investigational Treatment

Chemical Name: N-{4-[(6,7-dimethoxyquinolin-4-yl)oxy]phenyl}-N'-(4-fluorophenyl)cyclopropane-1,1-dicarboxamide, (2S)-hydroxybutanedioate

Exelixis internal number: XL184, cabozantinib

Exelixis will provide each investigator with adequate supplies of cabozantinib, which is supplied as 20 and 60 mg tablets. The components of the tablets are listed in Table B-1 in Appendix B.

Cabozantinib is an L-malate salt. In all studies except XL184-203, cabozantinib doses and capsule strengths are expressed based on the weight of the cabozantinib salt. However, in Study XL184-203, doses and capsule strengths are expressed based on the cabozantinib freebase equivalent weight. The difference between capsule strengths based on freebase or the L-malate salt weight (ie, < 2% based on correction for salt equivalent weights) is small relative to the variance in mean exposures in participants administered the same cabozantinib capsule dose (approximately 40% coefficient of variation for mean AUC), and is considered to be not clinically relevant. Cabozantinib tablets are expressed as freebase.

3.2 Dose, Schedule and Route

Participants will receive cabozantinib orally at a dose of 60 mg once daily. Cabozantinib should be swallowed intact, without dissolving or crushing. After fasting (with exception of water) for 2 hours, participants will take study treatment daily each morning with a full glass of water (minimum of 8 oz/ 240 mL) and continue to fast for 1 hour after each dose of study treatment. For missed doses, if it has been more than 12 hours since a scheduled dose, participants will be instructed not to take the missing dose. If participants vomit after taking their scheduled tablets, they will be instructed not to take another dose on this day and continue on their regular dosing schedule. If doses are withheld, the original schedule of assessments should be maintained when cabozantinib is restarted. The participant should be instructed to maintain the planned dosing schedule. The allowed dose reductions for cabozantinib are as below:

Table 3-1: Dose Modification Levels

Dose level	Cabozantinib dose
Starting dose	60 mg daily
-1	40 mg daily
-2	20 mg daily

In all subjects, dose reductions and delays to manage toxicity are allowed under the guidelines below.

3.3 Management of Adverse Events

The general adverse event profile of cabozantinib includes GI symptoms (such as nausea, vomiting, and diarrhea), fatigue, anorexia, PPE syndrome, skin rash, elevated ALT and AST, increased pancreatic enzymes with rare cases of pancreatitis, as well as side effects associated with inhibition of VEGF signaling such as thrombotic events (eg, PE and DVT), hypertension, proteinuria, hemorrhagic events, and rare cases of GI perforation and rectal/perirectal abscess. Arterial thromboembolism (TIA, MI) have been reported rarely.

3.3.1. Dose Reduction or Treatment Delay for Toxicity

In the absence of an unacceptable cabozantinib -related toxicity and/or disease progression, participants may continue treatment at the discretion of the investigator. Participants must be instructed to notify their physician immediately for any and all toxicities.

Guidelines for the management of AEs (ie, dose interruptions and dose reductions) are presented in the next sections. Each dose reduction of cabozantinib should be to one dose level lower that the current dose. Dose reductions of more than one dose level are acceptable if agreed to by the Investigator and the Sponsor. If study treatment of cabozantinib is restarted after being withheld or interrupted, the subject should be instructed not to make up the missed doses of cabozantinib.

The minimum dose of study treatment will be 20 mg PO qd. Subjects who cannot tolerate study treatment at 20 mg, or for whom treatment-related toxicity does not resolve to Grade ≤ 1 or baseline within 6 weeks after a dose interruption, will have study drug permanently discontinued.

The reason for treatment delay and reduced dose must be recorded on the CRF.

Dosing may need to be interrupted for AEs considered not related to cabozantinib if this is clinically indicated or if causality is initially uncertain. Study treatment may be resumed at the same dose (or a lower dose per investigator judgment) if the AE is determined not to be related to cabozantinib once the investigator determines that retreatment is clinically appropriate and the participant meets the protocol re-treatment criteria.

Dose interruptions for reason(s) other than toxicity, such as surgical procedures, may be allowed with sponsor approval. The acceptable length of interruption will depend on agreement between the investigator and the sponsor.

If study treatment is interrupted, the participant should be instructed not to make up the withheld doses, and the planned safety and tumor assessment schedule are to be maintained.

3.3.1.1 General Guidelines for Non-Hematologic and Hematologic Adverse Events

General guidelines for the management of non-hematologic and hematologic toxicities are provided in Table 3-2 Management of Study Treatment-Related Non-Hematologic Toxicities and in Table 3-3 Management of Study Treatment-Related Hematologic Toxicities.

As a general approach, it is suggested that all AEs be managed with supportive care when possible at the earliest signs of toxicity. For more specific guidelines on gastrointestinal AEs (diarrhea, nausea/vomiting, stomatitis/mucositis), hepatobiliary disorders, pancreatic disorders (lipase and amylase elevations), skin disorders (PPE), embolism and thrombus, hypertension, proteinuria, hemoptysis, abscess and gastrointestinal perforation and gastrointestinal fistula, refer to Sections 3.3.1.2 to 3.3.2.5 below. Guidance for the management of fatigue, anorexia, weight loss, wound healing, osteonecrosis of the jaw, endocrine disorders, eye disorders, musculoskeletal and connective tissue disorders, nervous system disorders, respiratory disorders and congenital, familial and genetic disorders can be found in the Investigators Brochure.

Table 3-2 Management of Study Treatment-Related Non-Hematologic Toxicities

CTCAE Version 3 Grade	Guidelines/Intervention
**Grade 1:**	Add supportive care as indicated. Continue study treatment at the current dose levels.
**Grade 2:**	
Grade 2 AEs considered related to study treatment that are subjectively tolerable or easily managed	Add supportive care as indicated. Continue study treatment at the current dose levels.
Grade 2 AEs considered related to study treatment that are intolerable to the participant or deemed unacceptable in the investigator's judgment; or are not easily managed or corrected	Dose reduce<br>• If the AE does not resolve to Grade ≤ 1 or baseline in 7 to 10 days or worsens at any time, cabozantinib dosing should then be interrupted. Then upon resolution to baseline or Grade ≤ 1, the reduced dose should be restarted.<br>• If the AE does resolve to Grade ≤ 1 or baseline without a dose interruption, continue the reduced dose.
**Grade 3:**	
Grade 3 AEs considered related to study treatment which occurred without optimal prophylaxis or which is easily managed by medical intervention or resolved quickly	• Interrupt study treatment and add supportive care as indicated<br>• For AEs that are easily managed (e.g., correction of electrolytes) with resolution to baseline or Grade ≤ 1 within 24 hours, treatment may be resumed at either the same dose or with a dose reduction at the discretion of the investigator unless this is a recurring event at which time the dose should be reduced<br>• For AEs that require supportive care, the dose should be held while supportive care is initiated and optimized. Then upon resolution of the AE to baseline or Grade ≤ 1, treatment should be restarted with a dose reduction. Note: if the investigator believes the likelihood of a reoccurrence of the same Grade 3 AE is small due to continued prophylaxis or other effective intervention, treatment may be resumed without a dose reduction and with very careful monitoring of the subject.
Grade 3 AEs considered related to study treatment that occurred despite optimal prophylaxis or is not easily managed by medical intervention	Interrupt study treatment until recovery to ≤ Grade 1 or baseline, and resume treatment with a dose reduction
**Grade 4:**	
Grade 4 AEs considered related to study treatment	Permanently discontinue study treatment unless determined that the participant is clearly deriving clinical benefit. In this case, upon recovery to Grade ≤ 1 or baseline, the participant may be re-treated at a reduced dose that is to be determined by the investigator and sponsor and only with approval by the sponsor.

Dose modifications or delays may occur in the setting of lower grade toxicity than defined above if the investigator believes that it is in the interest of the participant's safety. The Sponsor/Medical Monitor must then be informed.

Table 3-3: Management of Hematologic Toxicities

CTCAE Version 4 Grade	Intervention
**Neutropenia**	
Grade 3 neutropenia with documented infection<br>Grade 3 neutropenia ≥ 5 days<br>Grade 4 neutropenia	Interrupt cabozantinib treatment until resolution to Grade ≤ 1, and resume cabozantinib treatment at a reduced dose.
**Thrombocytopenia**	
Grade 3 thrombocytopenia with clinically significant bleeding or Grade 4 thrombocytopenia	Interrupt cabozantinib treatment until resolution to ≤ Grade 1, and resume cabozantinib treatment at a reduced dose.
**Febrile Neutropenia**	
Grade 3 febrile neutropenia	Interrupt cabozantinib treatment until recovery of ANC to Grade ≤ 1 and temperature to ≤ 38.0°C and resume cabozantinib treatment at a reduced dose.
Grade 4 febrile neutropenia	Permanently discontinue study treatment unless determined that the participant is clearly deriving clinical benefit. In this case, upon recovery to Grade ≤ 1 or baseline, the participant may be re-treated at a reduced dose that is to be determined by the investigator and sponsor and only with approval by the sponsor.

ANC, absolute neutrophil count.

Neutropenia: Grade 1 (LLN ≤ ANC < 1.5 × 10^9/L; Grade 2 (1 × 10^9/L ≤ ANC <1.5 x 10^9/L), Grade 3 (0.5 × 10^9/L ≤ ANC < 1 × 10^9/L), Grade 4 (ANC < 0.5 × 10^9/L).

Febrile Neutropenia CTCAE v4: Grade 3 (present); Grade 4 (Life-threatening consequences; urgent intervention indicated)

Thrombocytopenia: Grade 1 (<LLN – 75 x 10^9/L); Grade 2 (<75.0 – 50.0 x 10^9/L); Grade 3 (Platelet count ≤ 50-25 × 10^9/L); Grade 4 (Platelet count < 25 × 10^9/L).

3.3.1.2 Diarrhea, Nausea, Vomiting, Stomatitis, and Mucositis

Diarrhea

Subjects should be instructed to notify their physician immediately at the first signs of poorly formed or loose stool or an increased frequency of bowel movements. Administration of antidiarrheal agents is recommended at the first sign of diarrhea as initial management. Loperamide is recommended as standard first line therapy. Alternatively, diphenoxylate/atropine can be used. Additional agents to consider in participants with diarrhea that is refractory to the above include deodorized tincture of opium and octreotide (Benson et al. 2004). Some participants may require concomitant therapy with loperamide, diphenoxylate/atropine, and deodorized tincture of opium to control diarrhea. When combination therapy with antidiarrheal agents does not control the diarrhea to tolerable levels, a dose reduction and/or dose interruption of cabozantinib should be implemented as described in Table 3-2. In addition, general supportive measures should be implemented including continuous oral hydration, correction of fluid and electrolyte abnormalities, small frequent meals, and stopping lactose-containing products, high fat meals and alcohol. K, Mg, and Ca should be corrected to normal levels if they are abnormal.

Nausea and Vomiting
Anti-emetic agents along with supportive care are recommended as clinically appropriate at the first sign of nausea and vomiting. A dose reductions and/or dose interruption of cabozantinib may be required as described in Table 3-1 if antiemetic treatment and/or prophylaxis alone is not adequate.

Agents classified as having a high therapeutic index (such as 5-HT3 receptor antagonists, or NK-1 receptor antagonists) per ASCO or MASCC/ESMO guidelines for anti-emetics in oncology or dexamethasone are recommended (Hesketh et al. 2008, ASCO 2006; Roila et al, Annals of Oncology, 2010). Caution is recommended with the use of aprepitant or fosaprepitant and nabilone as cabozantinib exposure may be affected by concomitant administration because aprepitant and fosaprepitant are both inhibitors and inducers of CYP3A4, and nabilone is a weak inhibitor of CYP3A4.

Stomatitis and Mucositis
Preventive measures may include a comprehensive dental examination to identify any potential complications before study treatment is initiated. Appropriate correction of local factors should be instituted as indicated, such as modification of ill-fitting dentures and appropriate care of gingivitis. During treatment with cabozantinib, good oral hygiene and standard local treatments such as non-traumatic cleansing, and oral rinses (eg, with a weak solution of salt and baking soda) should be maintained. The oral cavity should be rinsed and wiped after meals, and dentures should be cleaned and brushed often to remove plaque. Local treatment should be instituted at the earliest onset of symptoms. When stomatitis interferes with adequate nutrition and local therapy is not adequately effective, dose reduction or temporary withholding of cabozantinib should be considered.

3.3.1.3 Hepatobiliary Disorders
Elevations of transaminases have also been observed during treatment with cabozantinib. In general, it is recommended that participants with elevation of ALT, AST, and/or bilirubin have more frequent laboratory monitoring of these parameters. If possible, hepatotoxic concomitant medications and alcohol should be discontinued in participants who develop elevated transaminases.

Since participants may enter the study with elevations of AST/ALT at baseline, the following guideline should be used for dose modifications:

Table 3-4: Management of ALT/AST Elevations

Transaminase elevation CTCAE v4.0	Intervention
**Subjects with AST and ALT less than or equal to the ULN at baseline**	
**Grade 1**	Continue study treatment with weekly monitoring of liver function tests (LFTs) for at least 4 weeks. Then resume the standard protocol-defined monitoring of LFTs.
**Grade 2**	Continue study treatment with at least twice weekly monitoring of LFTs for 2 weeks. Then weekly for 4 weeks. If LFTs continue to rise within Grade 2, interrupt study treatment. Then continue with at least weekly LFTs until resolution to Grade ≤ 1. Study treatment may then be resumed at a one-dose-level reduction of cabozantinib.
**Grade 3**	Interrupt study treatment and monitor with at least twice weekly LFTs until Grade ≤ 2. Then continue with at least weekly LFTs until resolution to Grade ≤ 1. Study treatment may then be resumed at a one-dose-level reduction of cabozantinib.
**Grade 4**	Discontinue study treatment permanently. LFTs should be monitored as clinically indicated, at least 2-3 times per week, until resolution to Grade ≤ 1. If the participant was unequivocally deriving clinical benefit, the participant may be able to resume treatment at a lower dose as determined by the investigator
**Subjects with AST or ALT above the ULN but ≤ 3.0 x ULN (i.e., Grade 1) at baseline**	
**≥ 1.5 fold transaminases increase (at least one of AST or ALT) and still Grade 1 or Grade 2**	Continue study treatment with at least twice weekly monitoring of LFTs for 4 weeks. If LFTs continue to rise, interrupt study treatment. Then continue with at least weekly LFTs until resolution to Grade ≤ 1. Study treatment may then be resumed at a one-dose-level reduction of cabozantinib
**≥ 1.5 fold transaminases increase (at least one of AST or ALT) and Grade 3**	Interrupt study treatment and monitor with at least twice weekly LFTs until Grade ≤ 2. Then continue with at least weekly LFTs until resolution to Grade ≤ 1. Study treatment may then be resumed at a one-dose-level reduction of cabozantinib.
**Grade 4**	Discontinue study treatment permanently. LFTs should be monitored as clinically indicated, at least 2-3 times per week, until resolution to Grade ≤ 1. If the participant was unequivocally deriving clinical benefit, the participant may be able to resume treatment at a lower dose as determined by the investigator.
**Subjects AST or ALT > 3.0 but ≤ 5.0 x ULN at baseline**	
**≥ 1.5 fold transaminases increase (at least one of AST or ALT) and still Grade 2 or Grade 3**	Interrupt study treatment and monitor with at least twice weekly LFTs until LFTs resolve to baseline and Grade ≤ 2. Study treatment may then be resumed at a one-dose-level reduction of cabozantinib.
**Grade 4**	Discontinue study treatment permanently. LFTs should be monitored as clinically indicated, at least 2-3 times per week, until resolution to Grade ≤ 1. If the participant was unequivocally deriving clinical benefit, the participant may be able to resume treatment at a lower dose as determined by the investigator.

Cabozantinib treatment should also be interrupted when transaminase increases are accompanied by progressive elevations of total bilirubin, and/or elevations of coagulation tests (eg, International Normalized Ratio [INR]). Monitoring of transaminases should be intensified (2–3 times per week) and cabozantinib should be held until the etiology of the abnormalities is determined and these abnormalities are corrected or stabilize at clinically acceptable levels (INR < 1.5 × ULN, total bilirubin < 1.5 × ULN, aminotransferases < 2.5 × ULN or baseline).

Subjects must have cabozantinib permanently discontinued if transaminase elevations are accompanied by evidence of impaired hepatic function (bilirubin elevation >2xULN), in the absence of evidence of biliary obstruction (i.e., significant elevation of alkaline phosphatase [ALP]) or some other explanation of the injury (e.g., viral hepatitis, alcohol hepatitis), as the combined finding (i.e., Hy's Law cases) represents a signal of a potential for the drug to cause severe liver injury.

All participants who develop isolated bilirubin elevations of Grade 3 should have study treatment held until recovered to Grade ≤ 1 or baseline (or lower). If this occurs within 6 weeks of the dosing delay, study treatment may continue at a reduced dose. In participants without biliary obstruction and Grade 4 bilirubin elevation, or with recurrence of Grade 3 bilirubin elevation after a dose reduction, study treatment must be discontinued.

Table 3-5: Management of Bilirubin Elevations

Bilirubin Elevation	Intervention
**Grade ≥ 3 Bilirubin**	Hold study treatment until recovered to Grade ≤ 1 or baseline (or lower). If recovery to this level occurs within 6 weeks of the dosing delay, study treatment may be continued at next lower dose.
**Re-occurrence of Grade 3 bilirubin after dose reduction; or Grade 4 bilirubin without biliary obstruction**	Discontinue study treatment

3.3.1.4 Pancreatic Conditions

Amylase and lipase elevations have been observed in clinical studies with cabozantinib. The clinical significance of asymptomatic elevations of enzymes is not known but in general have not been associated with clinically apparent sequelae. It is recommended that participants with lipase elevation and/or symptoms of pancreatitis have more frequent laboratory monitoring of lipase and/or amylase (2-3 times per week). Subjects with symptomatic pancreatitis should be treated with standard supportive measures.

Table 3-6: Asymptomatic Lipase or Amylase Elevations

**Asymptomatic Lipase or Amylase Elevations**	
Grade 1 or Grade 2	Continue at current dose level. More frequent monitoring is recommended
Grade 3	• Interrupt treatment<br>• Monitor lipase and amylase twice weekly<br>• Upon resolution to Grade ≤ 1 or baseline, cabozantinib may be restarted at the same dose or at a reduced dose provided that this occurs within 6 weeks.<br>• If retreatment following Grade 3 lipase or amylase elevation is at the same dose and Grade 3 elevations recur, then treatment must be interrupted again and till lipase and amylase levels have resolved to Grade ≤ 1 or baseline and retreatment must be at a reduced dose.
Grade 4	• Interrupt treatment<br>• Monitor lipase and amylase twice weekly<br>• Upon resolution to Grade ≤ 1 or baseline and if resolution occurred within 4 days, cabozantinib may be restarted at the same dose or a reduced dose. If resolution took more than 4 days, the dose must be reduced upon retreatment provided that resolution occurred within 6 weeks.<br>• If retreatment following Grade 4 lipase or amylase elevation is at the same dose and Grade 3 or 4 elevations recur, then treatment must be interrupted again until lipase and amylase have resolved to Grade ≤ 1 or baseline and retreatment must be at a reduced dose.

Table 3-7: Management of Symptomatic Pancreatitis

**Pancreatitis**	
Grade 2	• Interrupt treatment<br>• Monitor lipase and amylase twice weekly<br>• Upon resolution to Grade ≤ 1 or baseline, cabozantinib may be restarted at the same dose or at a reduced dose provided that this occurs within 6 weeks.<br>• If retreatment following Grade 2 pancreatitis is at the same dose and Grade 2 pancreatitis recurs, then treatment must be interrupted again and till resolution to Grade ≤ 1 or baseline and retreatment must be at a reduced dose.
Grade 3	• Interrupt treatment<br>• Monitor lipase and amylase twice weekly<br>• Upon resolution to Grade ≤ 1 or baseline , cabozantinib may be restarted at a reduced dose if resolution occurred within 6 weeks
Grade 4	Permanently discontinue treatment. However, if the participant was unequivocally deriving benefit from cabozantinib therapy, treatment may resume at a reduced dose per investigator judgment.

3.3.1.5 Skin Disorders

Hand-foot syndrome, skin rash (including blisters, erythematous rash, macular rash, skin exfoliation, dermatitis acneiform, and papular rash), pruritus, dry skin, and erythema have been reported in cabozantinib -treated subjects. Subjects with skin disorders should be carefully monitored for signs of infection (eg, abscess, cellulitis, or impetigo).

Early signs of hand-foot syndrome could be tingling, numbness, and slight redness or mild hyperkeratosis. Early manifestations include painful, symmetrical red and swollen areas on the palms and soles. The lateral sides of the fingers or periungual zones may also be affected. All participants on study should be advised on prophylactic skin care including the use of emollients, avoidance of exposure of hands and feet to hot water, protection of pressure-sensitive areas of hands and feet, and use of thick cotton gloves and socks to prevent injury and to keep the palms and soles dry. **Aggressive management of symptoms is recommended, including early dermatology referral. Dose reduction of study treatment to the next lower dose at the first sign of hand-foot syndrome is strongly recommended.**

In the case of study treatment-related skin changes (eg, rash, hand-foot syndrome), the investigator may request that additional assessments be conducted with the participant's consent. These assessments may include digital photographs of the skin changes and/or a biopsy of the affected skin and may be repeated until the skin changes resolve.

To estimate the body surface area for the purpose of grading skin toxicity per CTCAE the Modified Lund-Browder Chart should be used (Lund CC, Browder NC, 1944).

Table 3-8: Management of Hand Foot Skin Reaction

**Hand-Foot Skin Reaction and Hand Foot Syndrome (PPE)**	
No apparent toxicity	Prophylaxis with Ammonium lactate 12% cream (Amlactin®) twice daily OR heavy moisturizer (e.g. Vaseline) twice daily
Grade 1	Continue treatment at current dose if tolerable or reduce to the next lower dose if intolerable. Start urea 20% cream twice daily AND clobetasol 0.05% cream once daily. Assess participant at least weekly for changes in severity. Subjects should be instructed to notify investigator immediately if severity worsens. If severity worsens at any time or if there is no improvement after 2 weeks, proceed to the management guidelines for Grade 2 PPE
Grade 2	Reduce study treatment to next lower level and/or interrupt dosing. Start/continue urea 20% cream twice daily AND clobetasol 0.05% cream once daily. Pain control with NSAIDs/GABA agonists/narcotics. Assess participant at least weekly for changes in severity. Subjects should be instructed to notify investigator immediately if severity worsens. If severity worsens at any time (eg, peeling, blisters, bleeding, edema, or hyperkeratosis or affects self-care) or if there is no improvement after 2 weeks, proceed to the management guidelines for Grade 3 PPE. If the dose was reduced, then upon resolution to Grade 0 or Grade 1, treatment may continue at the reduced dose. If the dose was only interrupted but not reduced, then treatment may be restarted at one dose level lower.
Grade 3	Interrupt study treatment until severity decreases to Grade 1 or 0. Start/continue urea 20% cream twice daily AND clobetasol 0.05% cream once daily. Pain control with NSAIDs/GABA agonists/narcotics. Treatment may restart at one dose level lower when reaction decreases to Grade 1 or 0. Permanently discontinue participant from study if reactions worsen or do not improve within 6 weeks.

3.3.2 Embolism and Thrombosis

In clinical studies with cabozantinib, venous thrombotic events (DVT and PE) have been observed in less than 10% of subjects, and arterial thromboembolic events (MI and TIA) have been reported rarely. In addition, subjects with cancer have a significantly increased likelihood of developing thromboembolic complications (Agnelli et al, 2009).

Subjects who develop a PE and/or DVT should have study treatment interrupted until full anticoagulation is established with low molecular weight heparin (LMWH). (Full anticoagulation with warfarin is not permitted). Venous filters (e.g. vena cava filters) are not recommended due to the high incidence of complications associated with their use. Once a participant is fully anticoagulated, treatment can be restarted per investigator judgment at one dose lower. Subjects should permanently discontinue after a second thrombotic event. Although routine prophylactic anticoagulation is not necessary for all participants, prophylactic anticoagulation is allowed for individual participants at the discretion of the investigator.

Cabozantinib should be discontinued in participants who develop an acute MI or any other clinically significant arterial thromboembolic complication.

3.3.2.1 Hypertension

Hypertension is a relatively common complication of other VEGF-pathway inhibitors and has been observed in cabozantinib clinical studies.

Decisions to decrease or hold the dose of study treatment must be based on BP readings taken by a medical professional and must be confirmed with a second measurement at least 5 minutes following the first measurement. Other than for hypertension requiring immediate therapy, the presence of new or worsened hypertension should be confirmed at a second visit before taking new therapeutic action. Blood pressure should be monitored in a constant position visit to visit, either sitting or supine. Cabozantinib dosing should be interrupted in participants with severe hypertension (180 mm Hg systolic or 120 mm Hg diastolic; or sustained ≥ 160 mm Hg systolic or ≥ 110 diastolic) who cannot be controlled with medical interventions and discontinued in participants with hypertensive crises or hypertensive encephalopathy (Table 3-7).

Table 3-9: Management of Hypertension Related to Cabozantinib

Criteria for Dose Modifications	Treatment/cabozantinib Dose Modification
**Subjects not receiving optimized anti-hypertensive therapy**	
> 140 mm Hg (systolic) and < 160 mm Hg<br>OR<br>> 90 mm Hg (diastolic) and < 110 mm Hg	• Increase antihypertension therapy (i.e., increase dose of existing medications and/or add new antihypertensive medications)<br>• Maintain dose of cabozantinib<br>• If optimal antihypertensive therapy (usually to include 3 agents) does not result in blood pressure < 140 systolic or < 90 diastolic, dose of cabozantinib should be reduced.
≥ 160 mm Hg (systolic) and < 180 mm Hg<br>OR<br>≥ 110 mm Hg (diastolic) and < 120 mm Hg	• Reduce cabozantinib by one dose level.<br>• Increase antihypertension therapy (i.e., increase dose of existing medications and/or add new antihypertensive medications)<br>• Monitor participant closely for hypotension.<br>• If optimal antihypertensive therapy (usually to include 3 agents) does not result in blood pressure < 140 systolic or < 90 diastolic, dose of cabozantinib should be reduced further.
≥ 180 mm Hg (systolic) OR<br>≥ 120 mm Hg (diastolic)	• Interrupt treatment with cabozantinib Add new or additional anti-hypertensive medications and/or increase dose of existing medications.<br>• Monitor participant closely for hypotension.<br>• When SBP < 140 and DBP < 90, restart cabozantinib treatment at one dose level lower<br>• If optimal antihypertensive therapy (usually to include 3 agents) does not result in blood pressure < 140 systolic or < 90 diastolic, dose of cabozantinib should be reduced further.

BP, blood pressure, SBP systolic blood pressure, DBP diastolic blood pressure

NOTE: If SBP and DBP meet different criteria in table, manage per higher dose-modification criteria

3.3.2.2 Proteinuria

Proteinuria has been reported with approved drugs that inhibit VEGF pathways as well as with cabozantinib. Proteinuria diagnosed by dipstick should be quantified by a UPCR(mg/dL protein / mg/dL creatinine), When a UPCR exceeds 1, a repeat UPCR or a 24-hour urine protein and creatinine should be performed to confirm the result. Cabozantinib should be discontinued in subjects who develop nephrotic syndrome (proteinuria > 3.5 grams per day in combination with hypoalbuminemia, edema and hyperlipidemia) or any other relevant renal disease. Also, given the nephrotoxic potential of bisphosphonates, these agents should be used with caution in patients receiving treatment with cabozantinib. Details of management are described in Table 3-10.

Table 3-10 Management of Treatment Emergent Proteinuria

Urine Protein/Creatinine Ratio	Action To Be Taken
≤ 1	• No change in treatment or monitoring
> 1 and < 3.5	• No change in study treatment required<br>• Consider confirming with a 24-hour protein excretion within 7 days<br>• Repeat UPCR within 7 days and once every week. If UPCR is < 1 on two consecutive readings, then UPCR monitoring can revert to protocol specific time points. (The second reading is a confirmatory reading and can be done within 1 week of the first reading.).
≥ 3.5	• Hold cabozantinib immediately and confirm with 24 hour urine protein excretion.<br>• Evaluate for nephrotic syndrome. If present, discontinue cabozantinib treatment permanently, and monitor subject for resolution of nephrotic syndrome.<br>• If proteinuria of ≥ 3.5 g/24 hours is confirmed without diagnosis of nephrotic syndrome, continue to hold cabozantinib and monitor UPCR weekly. If UPCR decreases to < 1.5, restart cabozantinib at a reduced dose. Continue monitoring UPCR once every week until two consecutive readings are < 1, then revert to UPCR monitoring frequency specified in the protocol.

3.3.2.3 Hemorrhage

Hemorrhagic events have been reported with approved drugs that inhibit VEGF pathways as well as with cabozantinib. As preventive measures, participants should be evaluated for potential bleeding risk factors prior to initiating cabozantinib treatment and monitored for bleeding events with serial complete blood counts and physical examination while on study. Risk factors for hemorrhagic events may include (but may not be limited to) the following:

- Tumor lesions of the lung with cavitations or tumor lesions which invade, encase, or abut any major blood vessels; non-small cell lung cancer (NSCLC) with squamous cell differentiation is known for significant lung cavitations and centrally located tumors that may invade major blood vessels. The anatomic location and characteristics of tumor as well as the medical history should be carefully reviewed in the selection of participants for treatment with cabozantinib.
- Recent or concurrent radiation to the thoracic cavity
- Active peptic ulcer disease, ulcerative colitis, and other inflammatory GI diseases
- Underlying medical conditions which affect normal hemostasis (eg, deficiencies in clotting factors and/or platelet function, or thrombocytopenia)
- Concomitant medication with anticoagulants or other drugs which affect normal hemostasis

Based on the described predisposing risk factors for hemoptysis, many studies with antiangiogenic drugs exclude participants with NSCLC and squamous cell differentiation. Although enrollment of participants with NSCLC with squamous cell differentiation has been allowed on cabozantinib studies, cabozantinib studies exclude NSCLC participants with any of the following: tumors abutting, encasing, or invading a major blood vessel; cavitating lesions; history of clinically significant hemoptysis; or recent (within 3 months) radiation therapy to the thoracic cavity including brachytherapy unless radiation therapy targets bone metastasis.

Cabozantinib should be discontinued in participants with serious and life-threatening bleeding events or recent hemoptysis (≥ 0.5 teaspoon (2.5 ml) of red blood). Treatment with cabozantinib should be interrupted if less severe forms of clinically significant hemorrhage occur and may be restarted after the cause of hemorrhage has been identified and the risk of bleeding has subsided. Therapy of bleeding events should include supportive care and standard medical interventions.

Furthermore, participants who develop tumors abutting, encasing, or invading a major blood vessel or who develop cavitation of their pulmonary tumors while on study treatment must be discontinued from cabozantinib treatment.

3.3.2.4 Rectal and Perirectal Abscess

Rectal and perirectal abscesses have been reported, sometimes in participants with concurrent diarrhea. These should be treated with appropriate local care and antibiotic therapy. Cabozantinib should be held until adequate healing has taken place.

3.3.2.5 Wound healing and Surgery

VEGF inhibitors can cause wound healing complications and wound dehiscence which may occur even long after a wound has been considered healed. Therefore, surgical and traumatic wounds must have completely healed prior to starting cabozantinib treatment and be monitored for wound dehiscence or wound infection while the participant is being treated with cabozantinib.

Treatment with cabozantinib must be interrupted for any wound healing complication which needs medical intervention. Treatment with cabozantinib can be resumed once wound healing has occurred unless otherwise prohibited in specific protocols. Cabozantinib should be discontinued in participants with serious or chronic wound healing complications.

The appropriate dose hold interval prior to elective surgery to reduce the risk for wound healing complications has not been determined. In general, cabozantinib should be stopped at least 3 weeks (5 half lives) prior to elective surgery.

3.3.2.6 Endocrine Disorders

Prospective studies of markers of thyroid functions are currently ongoing in two single-agent studies to characterize the effects of cabozantinib on thyroid function. Preliminary data indicate that cabozantinib affects thyroid function tests (TFTs) in a high number of subjects. In Study XL184-203, 17 of 34 (50%) euthyroid subjects with castration-resistant prostate cancer (CRPC) developed abnormal thyroid-stimulating hormone (TSH) levels 6 weeks after initiation of cabozantinib treatment (6% showed TSH levels between 5 and 7 mU/L, 44% had

TSH > 7 mU/L). The median TSH level at week 6 was 5.2 mU/L (range, 0.02-29.7 mU/L). In a Phase 1 combination study of rosiglitazone and cabozantinib (XL184-008) to determine the potential for a clinically significant drug-drug interaction of cabozantinib on the CYP isozyme CYP2C8, subjects with advanced solid tumors (particularly renal cell carcinoma [RCC] and differentiated thyroid cancer [DTC]) are enrolled. Among 11 evaluable subjects, the AE of hypothyroidism was reported in 55% of subjects. Currently available data are insufficient to determine the cause of TFT alterations and its clinical relevance. Routine monitoring of thyroid function and assessments for signs and symptoms associated with thyroid dysfunction is recommended for participants treated with cabozantinib. Management of thyroid dysfunction (eg, symptomatic hypothyroidism) should follow accepted clinical practice guidelines.

Other endocrine disorders such as hypocalcemia and hyperglycemia, and associated laboratory changes, have been observed in less than 10% of participants. Monitoring with standard laboratory tests for endocrine disorders and clinical examination prior to initiation and during treatment with cabozantinib is recommended. Cabozantinib should be discontinued in participants with severe or life-threatening endocrine dysfunction.

3.3.2.7 Gastrointestinal perforation and GI fistula

Gastrointestinal perforation and GI fistula have been reported with approved drugs that inhibit VEGF pathways as well as with cabozantinib. To allow for early diagnosis, participants should be monitored for episodes of abdominal pain, especially if known risk factors for developing GI perforation or fistula (Turnage et al. 2008) are present. Such risk factors include (but may not be limited to) the following:

- Intra-abdominal tumor/metastases invading GI mucosa
- Active peptic ulcer disease, inflammatory bowel disease, ulcerative colitis, diverticulitis, cholecystitis or symptomatic cholangitis, or appendicitis
- History of abdominal fistula, GI perforation, bowel obstruction, or intra-abdominal abscess
- Prior GI surgery (particularly when associated with delayed or incomplete healing)

Complete healing following abdominal surgery or resolution of intra-abdominal abscess must be confirmed prior to initiating treatment with cabozantinib.

Additional risk factors include concurrent use of steroid treatment or non-steroidal anti-inflammatory drugs (Rodriguez et al. 2001, Straube et al. 2009). Constipation, consistent with symptoms of bowel obstruction, should be monitored and effectively managed. Discontinue cabozantinib and initiate appropriate management in participants who have been diagnosed with GI perforation or fistula.

3.3.2.8. Non-Gastrointestinal Fistula

Non-GI fistula formation has been reported with approved drugs that inhibit VEGF pathways as well as with cabozantinib. Radiation therapy has been identified as a possible predisposing risk factor for fistula formation in participants undergoing treatment with such agents (eg, bevacizumab). Radiation therapy to the thoracic cavity (including mediastinum) should be avoided within 3 months of starting treatment with cabozantinib. Non-GI fistula should be ruled out as appropriate in cases of onset of mucositis after start of therapy. Discontinue cabozantinib and initiate appropriate management in participants who have been diagnosed with a non-GI fistula.

3.3.2.9 QTc prolongation

If there is an increase in QTc interval to an absolute value > 500 msec, two additional EKGs should be performed within 30 minutes after the initial EKG with intervals no less than 3 minutes apart. (See section 5.5 for QTc interval calculations). If the change is noted and the participant is not in clinic, the participant should be notified immediately and brought to a medical facility to be evaluated as soon as possible with additional EKGs. If the average QTcF from three EKGs is > 500 msec, study treatment with cabozantinib must be withheld and the following actions should be taken:

1. Check electrolytes, especially potassium, magnesium and calcium. Correct abnormalities if present.
2. If possible, discontinue any QTc prolonging concomitant medications.
3. Repeat EKG triplets hourly until the average QTcF is ≤ 500 msec or otherwise determined by consultation with a cardiologist.

Exelixis should be notified immediately of any QTc prolongation event. Participants with QTc prolongation and symptoms must be monitored closely until the QTc elevation has resolved. Cardiology consultation is recommended for evaluation and management. Symptomatic participants must be treated according to standard clinical practice. No additional cabozantinib is to be given to the participant until after the event has resolved and the participant has been thoroughly evaluated. If any additional study treatment is given (e.g. after correction of electrolyte abnormalities and normalization of QTcF), it should be at the next reduced dose level.

3.4 Concomitant Medications and Therapies

3.4.1 Anticancer Therapy

If a participant requires additional systemic anticancer treatment, study treatment must be discontinued. Local intervention is discouraged unless medically unavoidable. Subjects receiving local intervention (eg, palliative radiation) are allowed to continue to receive study treatment at the investigator's discretion.

3.4.2 Other Medications

Subjects must be instructed to inform the investigators of the current or planned use or all other medications during the study (including prescription medications, vitamins, herbal and nutritional supplements, and over-the-counter medications). It is the responsibility of the investigator to ensure that details regarding all medications are documented.

Anti-emetics and anti-diarrheal medications should not be administered prophylactically prior to the first dose of cabozantinib. After the first dose of study treatment, at the discretion of the investigator or after the onset of symptoms, treatment (or prophylaxis) with anti-emetic and anti-diarrheal medications may be undertaken per standard clinical practice. Drugs used to control bone loss (eg, bisphosphonates, denosumab) are allowed if started prior to screening activities but may not be initiated or exchanged during the course of the study.

Colony stimulating factors (eg, erythropoietin and granulocyte colony-stimulating factors) and pain medications administered as dictated by standard practice are acceptable while the participant is enrolled in the study. However, colony stimulating factors should not be administered prophylactically prior to the first dose of study treatment.

No concurrent investigational agents are permitted.

3.4.3 Potential Drug Interactions

Cytochrome P450: Preliminary data from a clinical drug interaction study (Study XL184-008) show that clinically relevant steady-state concentrations of cabozantinib appear to have no marked effect on the AUC of co-administered rosiglitazone, a CYP2C8 substrate. Therefore, cabozantinib is not anticipated to markedly inhibit CYP2C8 in the clinic, and by inference, is not anticipated to markedly inhibit other CYP450 isozymes that have lower [I]/Ki values compared to CYP2C8 (ie, CYP2C9, CYP2C19, CYP2D6, CYP1A2, and CYP3A4). In vitro data indicate that cabozantinib is unlikely to induce cytochrome P450 enzymes, except for possible induction of CYP1A1 at high cabozantinib concentrations (30 μM).

Cabozantinib is a CYP3A4 substrate (but not a CYP2C9 or CYP2D6 substrate), based on data from in vitro studies using CYP-isozyme specific neutralizing antibodies. Preliminary results from a clinical pharmacology study, XL184-006, showed that concurrent administration of cabozantinib with the strong CYP3A4 inducer, rifampin, resulted in an approximately 80% reduction in cabozantinib exposure (AUC values) after a single dose of cabozantinib in healthy volunteers. Co-administration of cabozantinib with strong inducers of the CYP3A4 family (eg, dexamethasone, phenytoin, carbamazepine, rifampin, rifabutin, rifapentin, phenobarbital, and St. John's Wort) may significantly decrease cabozantinib concentrations. The chronic use of strong CYP3A4 inducers should be avoided. Other drugs that induce CYP3A4 should be used with caution because these drugs have the potential to decrease exposure (AUC) to cabozantinib. Selection of alternate concomitant medications with no or minimal CYP3A4 enzyme induction potential is recommended. In addition, caution must be used when discontinuing treatment with a strong CYP3A4 inducer in a participant who has been concurrently receiving a stable dose of cabozantinib, as this could significantly increase the exposure to cabozantinib.

Preliminary results from a clinical pharmacology study, XL184-007, showed that concurrent administration of cabozantinib with the strong CYP3A4 inhibitor, ketoconazole, resulted in a 33-39% increase in the cabozantinib exposure (AUC values) after a single dose of cabozantinib in healthy volunteers. Co-administration of cabozantinib with strong inhibitors of the CYP3A4 family (eg, ketoconazole, itraconazole, clarithromycin, indinavir, nefazodone, nelfinavir, and ritonavir) may increase cabozantinib concentrations. Grapefruit / grapefruit juice and Seville

oranges may also increase plasma concentrations of cabozantinib. Strong CYP3A4 inhibitors and other drugs that inhibit CYP3A4 should be used with caution because these drugs have the potential to increase exposure (AUC) to cabozantinib. Selection of alternate concomitant medications with no or minimal CYP3A4 enzyme inhibition potential is recommended. Because in vitro studies only assessed the metabolizing capacity of the CYP3A4, CYP2C9, and CYP2D6 pathways, the potential for drugs that inhibit/induce other CYP450 pathways (eg, CYP2C8, CYP2C19, CYP2B6, CYP1A2) to alter cabozantinib exposure is not known. Therefore, these drugs should be used with caution when given with cabozantinib.

Please refer to the Flockhart drug interaction tables for lists of substrates, inducers, and inhibitors of selected CYP450 isozyme pathways (Flockhart 2007; http://medicine.iupui.edu/clinpharm/ddis/table.aspx).

Protein Binding: Cabozantinib is highly protein bound (approximately 99.9%) to human plasma proteins. Therefore, highly protein bound drugs should be used with caution with cabozantinib because there is a potential displacement interaction that could increase free concentrations of cabozantinib and/or a co-administered highly protein-bound drug (and a corresponding increase in pharmacologic effect). Factors that influence plasma protein binding may affect individual tolerance to cabozantinib. Therefore, concomitant medications that are highly protein bound (eg, diazepam, furosemide, dicloxacillin, and propranolol) should be used with caution. Because warfarin is a highly protein bound drug with a low therapeutic index, administration of warfarin at therapeutic doses should be avoided in participants receiving cabozantinib due to the potential for a protein binding displacement interaction.

Other Interactions: A relative bioavailability study in dogs suggests that cabozantinib is unlikely to be affected by drugs that alter gastric pH. In vitro data suggest that cabozantinib is unlikely to be a substrate for P glycoprotein (P-gp), but it does appear to have the potential to inhibit the P-gp transport activity.

Additional details related to these overall conclusions are provided in the Investigators Brochure.

3.5 Compliance

Drug accountability and participant compliance will be assessed with drug dispensing and return records.

3.6 Study Drug Accountability

The investigator will maintain accurate records of receipt of all cabozantinib, including dates of receipt. In addition, accurate records will be kept regarding when and how much study treatment is dispensed and used by each participant in the study. Reasons for deviation from the expected dispensing regimen must also be recorded. At completion of the study, to satisfy regulatory requirements regarding drug accountability, all unused cabozantinib will be reconciled and destroyed in accordance with applicable state and federal regulations.

4 STUDY POPULATION

4.1 Inclusion Criteria

1. The participant has a histologic or cytologic diagnosis of a solid tumor (non-prostate, non-breast) that is metastatic and is refractory to or progressed (or relapsed) following standard therapies, or has disease for which no standard therapy exists. Presence of metastatic bone lesion(s) is required.
2. The participant is ≥ 18 years old on the day of consent.
3. The participant has an Eastern Cooperative Oncology Group (ECOG) performance status of 0 or 1.
4. The participant has organ and marrow function and laboratory values as follows:
   a. Absolute neutrophil count (ANC) ≥ 1500/mm3without colony stimulating factor support
   b. Platelets ≥ 100,000/mm3
   c. Hemoglobin ≥ 9 g/dL
   d. Bilirubin ≤ 1.5 × the upper limit of normal (ULN). For participants with known Gilbert's disease, bilirubin ≤ 3.0 mg/dL
   e. Serum albumin ≥ 2.8 g/dl
   f. Serum creatinine ≤ 1.5 × ULN or creatinine clearance ≥ 50 mL/min. For creatinine clearance estimation, the Cockcroft and Gault equation should be used:
      Male: CrCl (mL/min) = (140 - age) × wt (kg) / (serum creatinine × 72)
      Female: Multiply above result by 0.85
   g. Alanine aminotransferase (ALT) and aspartate aminotransferase (AST) ≤ 2.5 × ULN if no liver involvement, or ≤ 5 × ULN with liver involvement
   h. Lipase < 1.5 x the upper limit of normal (except for participants with adenocarcinoma of the pancreas)
   i. Urine protein/creatinine ratio (UPCR) ≤ 1
   j. Serum phosphorus ≥ LLN
   k. Ca, Mg, K within normal limits
5. The participant is capable of understanding and complying with the protocol requirements and has signed the informed consent document.
6. Sexually active participants (men and women) must agree to use medically accepted barrier methods of contraception (eg, male or female condom) during the course of the study and for 4 months after the last dose of study drug(s), even if oral contraceptives are also used. All participants of reproductive potential must agree to use both a barrier method and a second method of birth control.
7. Women of childbearing potential must have a negative pregnancy test at screening. Women of childbearing potential include women who have experienced menarche and who have not undergone successful surgical sterilization (hysterectomy, bilateral tubal ligation, or bilateral oophorectomy) or are not post-menopausal. Post-menopause is defined as:
   - Amenorrhea ≥ 12 consecutive months. Note: women who have been amenorrheic for 12 or more months are still considered to be of childbearing potential if the amenorrhea is possibly due to prior chemotherapy, antiestrogens, or ovarian suppression or any other reversible reason.

4.2 Exclusion Criteria

A participant who meets any of the following criteria is ineligible for the study:

1. The participant has received cytotoxic chemotherapy (including investigational cytotoxic chemotherapy) or biologic agents (eg, cytokines or antibodies) within 3 weeks, or nitrosoureas/ mitomycin C within 6 weeks before the first dose of study treatment.
2. The participant has received radiation therapy:
   a. to the thoracic cavity or gastrointestinal tract within 3 months of the first dose of study treatment.
   b. to bone or brain metastasis within 14 days of the first dose of study treatment
   c. to any other site(s) within 28 days of the first dose of study treatment
3. The participant has received radionuclide treatment within 6 weeks of the first dose of study treatment.
4. The participant has received prior treatment with a small molecule kinase inhibitor or a hormonal therapy (including investigational kinase inhibitors or hormones) within 14 days or five half-lives of the compound or active metabolites, whichever is longer, before the first dose of study treatment.
5. The participant has received any other type of investigational agent within 28 days before the first dose of study treatment.
6. The participant has not recovered to baseline or CTCAE ≤ Grade 1 from toxicity due to all prior therapies except alopecia and other non-clinically significant AEs.
7. The participant has a primary brain tumor.
8. The participant has active brain metastases or epidural disease
   (Note: Participants with brain metastases previously treated with whole brain radiation or radiosurgery or subjects with epidural disease previously treated with radiation or surgery who are asymptomatic and do not require steroid treatment for at least 2 weeks before starting study treatment are eligible. Neurosurgical resection of brain metastases or brain biopsy is permitted if completed at least 3 months before starting study treatment. Baseline brain scans are not required to confirm eligibility.)
9. The participant has prothrombin time (PT)/ International Normalized Ratio (INR) or partial thromboplastin time (PTT) test results at screening ≥ 1.3 × the laboratory ULN.
10. The participant requires concomitant treatment, in therapeutic doses, with anticoagulants such as warfarin or warfarin-related agents, heparin, thrombin or FXa inhibitors, or antiplatelet agents (eg, clopidogrel). Low dose aspirin (≤ 81 mg/day), low-dose warfarin (≤ 1 mg/day), and prophylactic low molecular weight heparin (LMWH) are permitted.
11. The participant has experienced any of the following within 3 months before the first dose of study treatment:
    a. clinically-significant hematemesis or lower gastrointestinal bleeding
    b. hemoptysis of ≥ 0.5 teaspoon (2.5 ml) of red blood
    c. any other signs indicative of pulmonary hemorrhage
12. The participant has radiographic evidence of cavitating pulmonary lesion(s) or tumor in contact with, invading or encasing major blood vessels

13. The participant has uncontrolled, significant intercurrent or recent illness including, but not limited to, the following conditions:
    a. Cardiovascular disorders including
        i. Congestive heart failure (CHF): New York Heart Association (NYHA) Class III (moderate) or Class IV (severe) at the time of screening
        ii. Uncontrolled hypertension defined as sustained BP > 140 mm Hg systolic, or > 90 mm Hg diastolic despite optimal antihypertensive treatment (BP must be controlled at screening)
        iii. Any of the following within 6 months before the first dose of study treatment:
            - unstable angina pectoris
            - clinically-significant cardiac arrhythmias
            - stroke (including TIA, or other ischemic event)
            - myocardial infarction
            - thromboembolic event requiring therapeutic anticoagulation (Note: participants with a venous filter (e.g. vena cava filter) are not eligible for this study)
    b. Gastrointestinal disorders particularly those associated with a high risk of perforation or fistula formation including:
        i. Any of the following at the time of screening
            - intra-abdominal tumor/metastases invading GI mucosa
            - active peptic ulcer disease
            - inflammatory bowel disease (including ulcerative colitis and Crohn's disease), diverticulitis, cholecystitis, symptomatic cholangitis or appendicitis
        ii. Any of the following within 6 months before the first dose of study treatment:
            (1) history of abdominal fistula
            (2) gastrointestinal perforation
            (3) bowel obstruction or gastric outlet obstruction
            (4) intra-abdominal abscess. Note: Complete resolution of an intra-abdominal abscess must be confirmed prior to initiating treatment with cabozantinib even if the abscess occurred more that 6 months ago.
        iii. GI surgery (particularly when associated with delayed or incomplete healing) within 28 days. Note: Complete healing following abdominal surgery must be confirmed prior to initiating treatment with cabozantinib even if surgery occurred more that 28 days ago.
    c. Other disorders associated with a high risk of fistula formation including PEG tube placement within 3 months before the first dose of study therapy or concurrent evidence of intraluminal tumor involving the trachea and esophagus.
    d. Other clinically significant disorders such as:
        i. active infection requiring systemic treatment
        ii. serious non-healing wound/ulcer/bone fracture
        iii. history of organ transplant
        iv. concurrent uncompensated hypothyroidism or thyroid dysfunction
        v. history of major surgery within 4 weeks or minor surgical procedures within 1 week before randomization
14. The participant is unable to swallow capsules or tablets

15. The patient has a corrected QT interval (QTcF) >500 ms within 28 days before randomization. If QTcF is $\geq$ 500 ms by Bazett formula, this must be confirmed by the Fridericia formula.

16. The participant is pregnant or breastfeeding.
17. The participant has a previously identified allergy or hypersensitivity to components of the study treatment formulation.
18. The participant is unable or unwilling to abide by the study protocol or cooperate fully with the investigator or designee.
19. The participant has had evidence within 2 years of the start of study treatment of another malignancy which required systemic treatment

4.3 Registration Procedures

General Guidelines for DF/HCC and DF/PCC Institutions

Institutions will register eligible participants with the DF/HCC Quality Assurance Office for Clinical Trials (QACT) central registration system. Registration must occur prior to the initiation of therapy. Any participant not registered to the protocol before treatment begins will be considered ineligible and registration will be denied.

A member of the study team will confirm eligibility criteria and complete the protocol-specific eligibility checklist.

Following registration, participants may begin protocol treatment. Issues that would cause treatment delays should be discussed with the Principal Investigator. If a participant does not receive protocol therapy following registration, the participant's protocol status must be changed. Notify the QACT Registrar of participant status changes as soon as possible.

Registration Process for DF/HCC and DF/PCC Institutions

The QACT registration staff is accessible on Monday through Friday, from 8:00 AM to 5:00 PM Eastern Standard Time. In emergency situations when a participant must begin treatment during off-hours or holidays, call the QACT registration line at 617-632-3761 and follow the instructions for registering participants after hours.

The registration procedures are as follows:

1. Obtain written informed consent from the participant prior to the performance of any study related procedures or assessments.
2. Complete the protocol-specific eligibility checklist using the eligibility assessment documented in the participant's medical/research record. **To be eligible for registration to the study, the participant must meet each inclusion and exclusion criteria listed on the eligibility checklist.**
3. Fax the eligibility checklist(s) and all pages of the consent form(s) to the QACT at 617-632-2295.
4. The QACT Registrar will (a) validate eligibility, (b) register the participant on the study, and (c) randomize the participant when applicable.

5. The QACT Registrar will send an email confirmation of the registration and/or randomization to the person initiating the registration immediately following the registration and/or randomization.

5 STUDY ASSESSMENTS AND PROCEDURES

5.1 Required Data

Table 5-1 Required Data

All study visits will have a +/- 3 day window

Please see section 5-9 for laboratory data.

Assessment	Screening within 28 days of first dose of study drug	Cycle 1 Cycle length = 28 days		Cycles 2-3		Cycles 4 and beyond*	End of Treatment
		C1D1	C1D15	CxD1	CxD15	CxD1	30-37days after last dose
Informed consent	X						
Demographics	X						
Medical History	X						
Physical exam	X	x	x	x	x	x	x
ECOG Performance Status	X	x	x	x	x	x	x
Vital signs	X	x	x	x	x	x	x
Urinalysis	X	x	x	x	x	x	x
PT/ or INR and PTT	X	x	x	x	x	x	x
Hematology	X	x	x	x	x	x	x
TSH, free T4	X						
Serum chemistry[a]	X	x	x	x	x	x	x
EKG[b]	X	x		x		x	x
Pregnancy test	X	Every two cycles					
Brain MRI or head CT	X						
Bone scan[c]	X			C2d1[c]			
PET scan[c]	X			C2d1[c]			
Tumor assessment Chest, abdominal, pelvic CT scan	X	Every 2 cycles (+/- 7 days)					
Concomitant medications	X	x	x	x	x	x	x
Adverse events	X	x	x	x	x	x	x
Bone biomarkers[d]	X	x	x	x		x	
Tumor sample for mutation and amplification testing[e]	X						
Pain Score[f]	X	x	x	x		x	
Analgesic medication diary[g]	X	x	x	x		x	
FACT-G	x	x	x	x		x	
Urine Creatinine and UPC Ratio [h]	X						

[a] Required serum chemistries include: Na, K, HCO3, Cl, BUN, Cr, Albumin, ALT, AST, total bilirubin, total protein, magnesium, phosphorus, ionized calcium or total and corrected calcium, glucose, amylase, lipase, LDH, CPK, ALP

[b] EKG will be done at baseline and on Day 1 of all cycles, after cabozantinib dose

[c] Bone scan and PET will be done at screening and then again at cycle 2 day 1, +/- 7 days. Note: we have obtained funding for bone scan and PET costs. This will be sufficient to cover for the first ten patients on study. More funding is being sought currently. If no additional funding is able to be gained, we will obtain bone scan and PET scan for the first ten patients only.

[d]See Table 5-2 for details

[e] Tumor samples will be tested for somatic mutations such as EGFR, KRAS, ALK, and for MET amplification. Genotyping/mutation testing is not an eligibility requirement. Testing will be completed only if tissue is available.

[f] Pain will be assessed by the Brief Pain Inventory (BPI) items #3 (worst pain over the last 24 hours by recall), #5 (average pain over the last 24 hours) and #9 (interference with daily activities and sleep). These questionnaires ask the participants to rate their pain based on an 11-point numerical rating system (NRS) ranging from 0 to 10, with 0 representing "No Pain," and 10 representing "Pain as Bad as You Can Imagine." The BPI is widely used in

contemporary pain studies and exhibits robust measurement properties including validity, reliability, and sensitivity (Atkinson et al. 2010).

[g]Subjects will also record all analgesic medications taken for pain on a paper pain medication diary provided by the site for the same 24 hour period. All analgesic medications are to be recorded in the diary, including but not limited to, narcotics, systemically administered corticosteroids, and NSAIDs.

h- Urine Creatinine and UPCR required at baseline and as needed while on treatment. Section 3.3.2.2 for details

5.2 Pre-Treatment Period

During the Pre-Treatment Period, participants are consented and screened for the study. Informed consent must be obtained before initiation of any clinical screening procedure that is performed solely for the purpose of determining eligibility for this study. Evaluations performed as part of routine care before informed consent can be considered as screening evaluations if done within the defined screening period, and if permitted by the site's institutional review board (IRB)/ ethics committee (EC) policies. Informed consent may be obtained more than 28 days before the first dose of study treatment. At informed consent, participants will be assigned a participant identifier. Subject identifiers are not to be re-assigned if a participant is determined to be ineligible, and participants are to maintain their original identifier if rescreening is required.

Study eligibility is based on meeting all of the study inclusion criteria and none of the exclusion criteria at screening and on Study Day 1 before study treatment administration.

For each participant, the Pre-Treatment Period ends upon receipt of the first dose of study treatment or final determination that the participant is ineligible for the study.

5.2.1 Screening Assessments

Informed consent must be obtained before any study-specific tests or evaluations are conducted. Please see required data table in Section 5.1 for full details of required screening assessments.

5.3 Study Treatment Period

Subjects are defined as enrolled upon receipt of the first dose of study treatment.

Each cycle is defined as 28 days.

During cycles 1-3, assessments will be made on day 1 and day 15.

In subsequent cycles, assessments will be made on day 1.

All assessments have a +/- 3 day window.

A detailed table of the required assessments while on study is shown in Table 5.1.

If the participant is unable to have a study assessment taken within the defined time window due to an event outside of his or her control (e.g., clinic closure, personal emergency, inclement weather, vacation), the assessment should be performed as close as possible to the required schedule.

Subjects should be instructed to inform the PI of any AEs. Subjects experiencing dizziness, sleepiness, or other symptoms that could influence alertness or coordination should be advised not to drive or operate other heavy machinery.

Subjects may receive study treatment until the earlier of progressive disease (PD) or unacceptable toxicity. Regular tumor assessments should be performed according to the guidelines in Section 5.1 to determine if PD is present.

The Treatment Period ends when a participant receives his or her last dose of study treatment; the participant then enters the Post-Treatment Period.

5.4 Post-Treatment Period

Subjects will return to the study site between 30 – 37 days after the last dose of cabozantinib for an end-of-treatment assessment. Laboratory and physical examinations will be performed. Remaining study treatment will be returned by the participant, and treatment compliance will be documented. Additional follow-up will occur for participants with AEs related to study treatment that are ongoing at the time of this visit, and for participants with SAEs related to study treatment that occur after the time of this visit.

All SAEs that are ongoing 30 days after the last dose of study treatment, and AEs assessed Grade 3 or 4 that led to study treatment discontinuation that are ongoing 30 days after the last dose of study treatment, are to be followed until:

- the AE has resolved, or;
- the AE has improved to Grade 2 or lower, or;
- the investigator determines that the event has become stable or irreversible.

This requirement also applies to related SAEs that occur > 30 days after last dose of study treatment.

The status of all other AEs that are ongoing 30 days after the last dose of study treatment will be documented as of the Post-Treatment Follow-Up Visit.

5.5 Electrocardiogram (ECG) Assessments

ECG assessments will be performed with standard 12-lead ECG equipment according to standard procedures. At any time point, if there is an increase in QTc interval to an absolute value > 500 msec, using the Bazett formula, the QTc must be calculated using the Fridericia formula as well.

$$QTcF = \frac{QT}{RR^{1/3}}$$

If the QTc by the Fridericia is > 500 msec, two additional ECGs should be performed approximately 2 minutes apart, within 30 minutes. If the average QTc interval calculated by the Fridericia formula from the three ECGs is > 500 msec, study treatment must be withheld and a cardiology consultation is recommended for evaluation and participant management. Study treatment may only be continued if the QTc resolves to 500 msec or less and per investigator judgment that continued treatment is appropriate. Abnormalities in the ECG that lead to a change in participant management (eg, dose reduced or withheld, requirement for additional medication or monitoring) or result in clinical signs and symptoms are considered clinically significant for the purposes of this study and will be recorded on the AE CRF. If values meet criteria defining them as serious, they must be reported as SAEs (Section 6.1.2).

5.6 Vital Signs
Vital signs (body temperature, respiratory rate, and blood pressure and pulse) will be conducted at regular intervals. Blood pressure and pulse will be measured after the participant has been sitting for at least 5 minutes.

When vital signs are scheduled at the same time as blood draws, the blood draws will be obtained at the scheduled time point, and the vitals will be obtained as close to the scheduled blood draw as possible.

5.7 Physical Examinations
A physical examination will include assessments of general appearance, skin, HEENT, thorax/lungs, cardiovascular, abdominal, genitourinary, musculoskeletal and neurological findings. Any pertinent findings should be documented either in the participant's medical history (if determined to be prior to the first dose of cabozantinib) or as an AE (if new or worsening after the first dose of cabozantinib).

5.8 Pain/Analgesic Assessment and QOL assessment
Assessment of pain will be self-collected by each participant using the Brief Pain Inventory (BPI) items #3 (worst pain over the last 24 hours by recall), #5 (average pain over the last 24 hours) and #9 (interference with daily activities and sleep). These questionnaires ask the participants to rate their pain based on an 11-point numerical rating system (NRS) ranging from 0 to 10, with 0 representing "No Pain," and 10 representing "Pain as Bad as You Can Imagine." The BPI is widely used in contemporary pain studies and exhibits robust measurement properties including validity, reliability, and sensitivity (Atkinson et al. 2010).

Subjects will also record all analgesic medications taken for pain on a paper pain medication diary provided by the site for the same 24 hour period.

The FACT-G will be used for quality of life assessment and will be obtained at the time of clinic visit as per the scheduled in Table 5-1.

5.9 Laboratory Assessments

Local laboratories will perform all laboratory tests, and results will be provided to the investigator. Blood and urine samples for hematology, serum chemistry, and urinalysis will be prepared using standard procedures. Laboratory results will be reviewed by the investigator for clinical significance. Laboratory panels are composed of the following:

**Hematology**		
• white blood cell (WBC) count with differential (including at minimum: neutrophils, basophils, eosinophils, lymphocytes, monocytes)	• hematocrit<br>• platelet count<br>• red blood cell (RBC) count<br>• hemoglobin<br>• PT/INR and PTT	
**Serum chemistry**		
• albumin<br>• alkaline phosphatase (ALP)<br>• amylase<br>• ALT<br>• AST<br>• bicarbonate<br>• blood urea nitrogen (BUN)<br>• chloride	• creatinine<br>• glucose<br>• ionized calcium or total and corrected calcium<br>• lactate dehydrogenase<br>• lipase	• magnesium<br>• phosphorus<br>• potassium<br>• sodium<br>• total bilirubin<br>• total protein
**Urinalysis**		
• appearance<br>• color<br>• pH<br>• specific gravity<br>• ketones<br>• protein	• glucose<br>• bilirubin<br>• nitrite<br>• creatinine<br>• urobilinogen	• occult blood (microscopic examination of sediment will be performed only if the results of the urinalysis dipstick evaluation are positive)
**Other (at screening or as needed)**		
• TSHand free T4<br>• Pregnancy text (urine or serum) for women of child-bearing potential		• urine protein/creatinine ratio (UPCR) (if needed, see Section 3.3.2.2.)<br>• 24 hour urine collection for protein (if needed, see Section 3.3.2.2.)

INR, International Normalized Ratio; PT, prothrombin time; PTT partial thromboplastin time; TSH, thyroid stimulating hormone

Abnormalities in clinical laboratory tests that lead to a change in participant management (eg, dose delayed (withheld) or reduced, requirement for additional medication, treatment or monitoring) are considered clinically significant for the purposes of this study, and will be recorded on the Adverse Events CRF. If laboratory values constitute part of an event that meets criteria defining it as serious, the event (and associated laboratory values) must be reported as an SAE.

Bone biomarker assessments will be performed by the Clinical Core Research Laboratory at MGH. Blood draws will be performed according to study protocol. The samples will be delivered to the lab between 9am and 4pm by the study staff. The CLR core will receive specimens and accession them under unique laboratory barcode identifiers. The laboratory will perform testing and other services under CLIA Certification # 22D0706493 [Expiration date 02/27/2013]. Table 5-2 shows the bone biomarkers to be tested.

Table 5-2 Bone biomarkers

TEST	Method	VOLUME FOR TESTING	TUBE TYPE
C-telopeptide	LabCorp- ELISA	100 uL	Red top tube
Bone Alkaline phosphatase	CLR- micro elctrochemiluminescence	50 uL	Red top tube
Osteocalcin	CLR- micro elctrochemiluminescence	250 uL	Red top tube
Serum N-telopeptide	LabCorp- ELISA	300 uL	Red top tube
Serum Procollagen type 1 N-terminal peptide	LabCorp- ELISA	300 uL	Red top tube
Urinary N-telopeptide	LabCorp- ELISA	3 mL	No-preservative tube

In addition, routine clinical tumor genotyping and MET FISH will be obtained on all patients with available tumor tissue, if it has not already been performed. Samples will be processed in batches and the results may not be immediately available.

5.10 Tumor Assessment

5.10.1 Routine Tumor Assessment

Tumor response should be assessed every 8 weeks by CT scans. Subjects continuing to show benefit, (CR, PR, SD or clinically-determined benefit) may continue on study. Subjects with PD or clinical deterioration should have their treatment discontinued, and they should enter the post-treatment phase of the study. Note that the decision to continue on study or not will be made based on these CT scan results or clinical determination on the part of the investigator, and not on any results of bone imaging. The same method for tumor assessment should be employed at every assessment.

5.10.2 Bone imaging

Bone scan and PET scan will be obtained at screening and then again at Cycle 2 day 1, ± 7 days. Note: we have obtained funding for bone scan and PET costs; this will be sufficient to cover for the first ten patients on study; more funding is being sought currently. If no additional funding is able to be gained, we will obtain bone scan and PET scan for the first ten patients only.

6 SAFETY

6.1 Adverse Events and Laboratory Abnormalities

6.1.1 Adverse Events

An AE is any untoward medical occurrence in a patient or clinical investigation participant who has been enrolled in a clinical study and who may have been given an investigational product, regardless of whether or not the event is assessed as related to the study treatment. An AE can therefore be any unfavorable and unintended sign (including an abnormal laboratory finding), symptom, or disease temporally associated with the use of an investigational product, regardless of whether or not the event is assessed as related to the investigational product. Pre-existing medical conditions that worsen during a study should be recorded as AEs. Abnormal laboratory values, electrocardiogram (ECG) findings, or vital signs are to be recorded as AEs if they meet the criteria described in Section 6.2.1.

All untoward events that occur after informed consent through 30 days after the last dose of study treatment are to be recorded by the investigational site. This requirement includes AEs from unscheduled as well as scheduled visits.

Assessment of the relationship of the AE to the study treatment by the investigator is based on the following two definitions:

- Not Related: A not-related AE is defined as an AE that is not associated with the study treatment and is attributable to another cause.
- Related: A related AE is defined as an AE where a causal relationship between the event and the study treatment is a reasonable possibility. A reasonable causal relationship is meant to convey that there are facts (eg, evidence such as dechallenge/ rechallenge) or other clinical arguments to suggest a causal relationship between the AE and study treatment.

6.1.2 Serious Adverse Events

The SAE definition and reporting requirements are in accordance with the International Conference of Harmonisation (ICH) Guideline for Clinical Safety Data Management: Definitions and Standards for Expedited Reporting, Topic E2A.

An SAE is defined as any untoward medical occurrence that at any dose:

1. Results in death.
2. Is immediately life-threatening (ie, in the opinion of the investigator, the AE places the participant at immediate risk of death; it does not include a reaction that, had it occurred in a more severe form, might have caused death).
3. Requires inpatient hospitalization or results in prolongation of an existing hospitalization.
4. Results in persistent or significant disability or incapacity.

   Note: The term “disability” refers to events that result in a substantial disruption of a participant’s ability to conduct normal life function.
5. Is a congenital anomaly or birth defect.

6. Is an important medical event (IME). Note: The term "important medical event" refers to an event that, based upon appropriate medical judgment, may not be immediately life-threatening or result in death or hospitalization, but may jeopardize the participant or require intervention to prevent one of the other serious outcomes listed under the definition of Serious Adverse Event. Examples of IMEs include intensive treatment in an emergency room or at home for allergic bronchospasm; blood dyscrasias, or convulsions that do not result in hospitalization; or development of product dependency or product abuse.

Events **not** considered to be serious adverse events are hospitalizations for:

- routine treatment or monitoring of the studied indication, not associated with any deterioration in condition, or for elective procedures
- Abnormal lab values that do not require treatment
- treatment planned before signing informed consent for a pre-existing condition that did not worsen
- emergency outpatient treatment for an event not fulfilling the serious criteria outlined above and not resulting in inpatient admission
- respite care
- death due to progression of disease

6.1.3 Serious Adverse Event Reporting

As soon as an investigator becomes aware of an AE that meets the definition of 'serious,' this should be documented to the extent that information is available.

- This report must be submitted by the study site to the DF/HCC IRB in accordance with OHRS policy.

- This report must be submitted by the study site to Exelixis or designee within 24 hours, even if it is not felt to be drug related. Email: drugsafety@exelixis.com; Fax 650-837-7392
- The investigator agrees to provide supplementary information requested by the Exelixis Drug Safety personnel or designee.
- Pregnancy, although not itself an SAE, should also be reported on an SAE form or pregnancy form and be followed up to determine outcome, including spontaneous or voluntary termination, details of birth, and the presence or absence of any birth defects or congenital abnormalities.

6.1.4 Regulatory Reporting

All serious unexpected adverse drug reactions (unexpected related SAEs) must be reported to the Food and Drug Administration (FDA) by the investigator as required by 21 CFR 312.32.

- These reports are to be filed utilizing the Form FDA 3500A (MedWatch Form).

The final MedWatch Form must be submitted by the study site to Exelixis at the time of submission to the FDA to allow Exelixis time to cross-report to Exelixis' IND. Email: drugsafety@exelixis.com; Fax 650-837-7392.

6.2 Other Safety Considerations

6.2.1 Laboratory Data

All laboratory data required by this protocol and any other clinical investigations should be reviewed. Any abnormal value that leads to a change in participant management (eg, dose reduction or delay or requirement for additional medication or monitoring) or that is considered to be of clinical significance by the investigator should be reported as an AE or SAE as appropriate.

6.2.2 Pregnancy

If a participant becomes pregnant during the study, she will be taken off study treatment and will be followed through the end of her pregnancy. The investigator must inform Exelixis of the pregnancy. Forms for reporting pregnancies will be provided to the study sites upon request. The outcome of a pregnancy (for a participant or for the partner of a participant) and the medical condition of any resultant offspring must be reported to Exelixis or designee. Any birth defect or congenital anomaly must be reported as an SAE, and any other untoward events occurring during the pregnancy must be reported as AEs or SAEs, as appropriate.

6.2.3 Medication Errors/ Overdose

Any study drug administration error or overdose that results in an AE, even if it does not meet the definition of serious, requires reporting within 24 hours to Exelixis or designee.

6.2.4 Follow-Up of Adverse Events

Any related SAEs or any AEs assessed as related that led to treatment discontinuation, including clinically significant abnormal laboratory values that meet these criteria, ongoing 30 days after the last dose of study treatment must be followed until either resolution of the event or determination by the investigator that the event has become stable or irreversible. This follow-up guidance also applies to related SAEs that occur > 30 days after the last dose of study treatment. The status of all other continuing AEs will be documented as of 30 days after the last dose of study treatment.

7 STATISTICAL CONSIDERATIONS

7.1 Analysis Population

The analysis population will consist of all evaluable participants who enroll in the study.

7.1.1 Safety Population

The safety population will consist of all participants who receive any amount of study treatment.

7.2 Safety Analysis

Safety will be assessed by evaluation of AEs. All safety analyses will be performed using the safety population.

7.2.1 Adverse Events

Adverse event terms recorded on the CRFs will be mapped to preferred terms using the Medical Dictionary for Regulatory Activities (MedDRA). Seriousness, severity/ grade and relationship to study treatment will be assessed by the investigator. Severity/ grade will be defined by the National Cancer Institute (NCI) CTCAE v4.0. Listings of AEs will be provided.

7.3 Sample Size

The total sample size will be approximately 38 participants.

A two-stage phase II design will used, with an interim analysis and an early stopping rule for inactivity. The minimax design will be used. The first stage will include 19 evaluable patients by bone biomarker results. Based upon the definition of evaluable added in the previous amendment, it was necessary to increase the overall accrual number to 28 to the first part of this phase II study. If at least 5 of 19 evaluable patients achieve a bony response to treatment, defined as ≥ 40% decrease in urinary Ntx, serum Ntx, or serum Ctx at week 8, enrollment will proceed with 10 additional patients. The underlying assumption is that the regimen will be of interest if the proportion of patients achieving the ≥ 40% decrease in bone biomarker is ≥ 45%, and not of interest if the proportion achieving the endpoint is ≤ 20%.. This design will guarantee an overall significance level of 0.05 with power of 90%.

Bony response is defined as ≥ 40% decrease in any of the following biomarkers: urinary Ntx, serum Ntx, or serum Ctx. The ≥ 40% decrease was chosen as the cutoff as this degree of change has previously been used as a clinically meaningful endpoint in a study of a tyrosine kinase inhibitor (dasatinib) in prostate cancer [Yu 2009]. Previous reports have also shown that this level of decrease correlated with reduction in risk of death regardless of baseline uNtx level [Lipton 2008]. The recommendations for the null and alternative hypotheses are based on data from a randomized discontinuation study in breast cancer, where approximately 7/16 (~45%) patients had serum NTx reductions of ≥ 40% [Tolaney 2011], with serum CTx trending similarly, and this range of effect in other cancers would be considered of interest. We have chosen a composite of the urinary Ntx, serum Ntx, and serum Ctx biomarkers to define the bony response, due to a combination of the published data cited above as well as the experience with cabozantinib and serum Ntx and Ctx in prior studies.

Analysis of secondary endpoints:

Analysis of secondary endpoints are considered exploratory and will be descriptive only.

Skeletal related events are defined as any of the following events: (1) pathologic fracture; (2) cord compression; (3) radiation or surgery to bone for metastatic disease; (3) hypercalcemia. These events will be collected as adverse events prospectively, and the occurrence of any one of these events will count as having an SRE. Time to SRE will measured from date of registration on study to date of first SRE. The Kaplan-Meier method will be used to estimate time to SRE.

Quality of life is measured by the FACT-G questionnaire and pain/analgesic scale. Analysis of variance with repeated measures (ANOVA) will be used to estimate the changes in QOL scores during therapy.

Tumor response rate will be measured using CT imaging at baseline and every two cycles on cabozantinib. RECIST v1.1 criteria will be used to determine tumor response.

Tumor genotypes and MET amplification will be assessed for association with RECIST tumor response by Fisher's exact test.

Analysis of bone scan and PET scan: A dedicated radiologist will read the baseline and on-treatment bone and PET-CT scans on the patients enrolled in the study. There is currently no clear consensus on the best way to interpret bone imaging findings for metastatic disease. The criteria described by the UICC and WHO for assessment of disease response in bone are based on plain radiography and bone scan assessments only. A newer proposed response assessment algorithm from MD Anderson relies on plain radiography, CT, or MRI and uses bone scan to support the other findings, but does not incorporate PET [Hamaoka et al 2004]. We recognize up front that there is no established gold standard in the measurement of bony metastases. In our assessments, a dedicated radiologist blinded to bone biomarker or RECIST data will read the PET scans for SUV uptake and bone scans for response on a scale of 1-5. These responses will be correlated with bone biomarker response and CT response of extraosseous metastases by RECIST criteria using Pearson chi-squared tests (for categorical variables) and Kruskal-Wallis tests (for continuous variables), where appropriate. We will assess rates of discordance between bone scan and PET-CT scan and attempt to resolve these by comparison with RECIST response.

8 DATA QUALITY ASSURANCE

Accurate and reliable data collection will be ensured by verification and crosscheck of the CRFs against the investigator's records by the study monitor (source document verification) and by the maintenance of a drug–dispensing log by the investigator.

9 ETHICAL ASPECTS

9.1 Local Regulations

The study must fully adhere to the principles outlined in "Guideline for Good Clinical Practice" (GCP) ICH E6 Tripartite Guideline (January 1997). The investigator will ensure that the conduct of the study complies with the basic principles of GCP as outlined in the current version of 21 Code of Federal Regulations, subpart D, Part 312, "Responsibilities of Sponsors and Investigators" Part 50, "Protection of Human Subjects" and Part 56, "Institutional Review Boards."

9.2 Informed Consent

It is the responsibility of the investigator, or a person designated by the investigator, to obtain written informed consent from each participant participating in this study after adequate explanation of the aims, methods, anticipated benefits, and potential hazards of the study. In the case where the participant is unable to read, an impartial witness should be present during the entire informed consent discussion. After the participant has orally consented to participation in the trial, the witness's signature on the form will attest that the information in the consent form was accurately explained and understood.

The CRF for this study contains a section for documenting informed participant consent, and this must be completed appropriately. If new safety information results in significant changes in the risk/ benefit assessment, the consent form should be reviewed and updated as necessary. All participants (including those already being treated) should be informed of the new information, should be given a copy of the revised form, and should give their consent to continue in the study.

9.3 Institutional Review Board/ Ethics Committee

This study is being conducted under a United States Investigational New Drug application or other Clinical Trial Application, as appropriate. This protocol (and any modifications) and appropriate consent procedures must be reviewed and approved by an IRB/ EC. This board must operate in accordance with current local, regional, and federal regulations. The investigator will send a letter or certificate of IRB/ EC approval to Exelixis (or designee) before participant enrollment and whenever subsequent modifications to the protocol are made.

10 CONDITIONS FOR MODIFYING THE PROTOCOL

Protocol modifications may be made and will be prepared, reviewed, and approved by representatives of the investigator.

All protocol modifications must be submitted to the IRB/ EC for information and approval in accordance with local requirements and to regulatory agencies if required. Approval must be obtained before any changes can be implemented, except for changes necessary to eliminate an immediate hazard to study participants or those that involve only logistical or administrative aspects of the trial (eg, change in monitor or change of telephone number).

11 CONDITIONS FOR TERMINATING THE STUDY

Exelixis reserves the right to terminate the study, and investigators reserve the right to terminate their participation in the study, at any time. Should this be necessary, Exelixis and the investigator will arrange the procedures on an individual study basis after review and consultation. In terminating the study, Exelixis and the investigator will ensure that adequate consideration is given to the protection of the participants' interests.

12 STUDY DOCUMENTATION AND RECORDKEEPING

12.1 Investigator's Files and Retention of Documents

The investigator must maintain adequate and accurate records to enable the conduct of the study to be fully documented and the study data to be subsequently verified. These documents should be classified into two separate categories as follows: (1) the investigator's study file, and (2) participants' clinical source documents.

The investigator's study file will contain the protocol and protocol amendments, CRFs, query forms, IRB/ EC and governmental approvals with correspondence, sample informed consent, drug records, staff curriculum vitae and authorization forms, and other appropriate documents and correspondence.

Subjects' clinical source documents include the participants' hospital/ clinic records; physicians' and nurses' notes; the appointment book; original laboratory, ECG, electroencephalogram, X-ray, pathology and special assessment reports; signed informed consent forms; consultant letters; and participant screening and enrollment logs.

The investigator must keep these documents on file for at least 2 years after the marketing application approval date for the study treatment and for the indication being investigated or for 2 years after the investigation is discontinued and the FDA is notified. After that period, the documents may be destroyed subject to local regulations with prior written permission from Exelixis. If the investigator wants to assign the study records to another party or move them to another location, Exelixis must be notified in advance.

If the investigator cannot guarantee the archiving requirement at the study site for any or all of the documents, special arrangements must be made between the investigator and Exelixis to store these in a sealed container outside of the study site so that they can be returned sealed to the investigator in case of a regulatory audit. When source documents are required for the continued care of the participant, appropriate copies should be made for storing outside of the study site.

12.2 Source Documents and Background Data

Upon request, the investigator will supply its licensees and collaborators with any required background data from the study documentation or clinic records. This is particularly important when CRFs are illegible or when errors in data transcription are suspected. In case of special problems or governmental queries or requests for audit inspections, it is also necessary to have access to the complete study records, provided that participant confidentiality is protected.

12.3 Audits and Inspections

The investigator should understand that source documents for this study should be made available, after appropriate notification, to qualified personnel from the Exelixis Quality Assurance Unit (or designee) or to health authority inspectors. The verification of the CRF data must be by direct inspection of source documents.

12.4 Case Report Forms

For enrolled participants, all and only data from the procedures and assessments specified in this protocol and required by the CRFs should be submitted on the appropriate CRF. Data from some procedures required by the protocol, such as physical examinations and laboratory results, will be recorded only on the source documents and will not be transcribed to CRFs. Additional procedures and assessments may be performed as part of the investigator's institution or medical practice standard of care and may not be required for CRF entry.

For each participant enrolled, the CRF (paper or electronic) must be completed and signed by the PI or authorized delegate from the study staff.

All paper forms should be typed or filled out using indelible ink and must be legible. Errors should be crossed out but not obliterated, the correction inserted, and the change initialed and dated by the investigator or his or her authorized delegate.

The investigator should ensure the accuracy, completeness, legibility, and timeliness of the data in the CRF and in all required reports.

13 MONITORING THE STUDY

It will be the monitor's responsibility to inspect the CRFs at regular intervals throughout the study to verify both adherence to the protocol and the completeness, consistency, and accuracy of the data being entered on them. The monitor should have access to laboratory test reports and other participant records needed to verify the entries on the CRF. The investigator (or designee) must agree to cooperate with the monitor to ensure that any problems detected in the course of these monitoring visits are resolved.

14 CONFIDENTIALITY OF TRIAL DOCUMENTS AND SUBJECT RECORDS

The investigator must assure that participants' anonymity will be maintained and that their identities are protected from unauthorized parties. On CRFs or other documents submitted to Exelixis or designees, participants should be identified by identification codes and not by their names. The investigator should keep a participant enrollment log showing codes, names, and addresses. The investigator should maintain documents not for submission to Exelixis or designees (eg, participants' written consent forms) in strict confidence.

All tumor scans, research samples, photographs, and results from examinations, tests, and procedures may be sent to Exelixis and its partners or designees for review.

15 PUBLICATION OF DATA AND PROTECTION OF TRADE SECRETS

The Principal Investigator (Protocol Chair) holds the primary responsibility for publication of the study results; provided that the PI will provide any such publication to Exelixis, Inc. for review at least sixty (60) days prior to submission and also comply with any provisions regarding publication as are agreed to between the PI's institution (eg, institution name.) and Exelixis, Inc. in the Clinical Trial Agreement related to this study. The results will be made public within 24 months of the end of data collection. However, if a report is planned to be published in a peer-reviewed journal, then that initial release may be an abstract that meets the requirements of the International Committee of Medical Journal Editors. In any event, a full report of the outcomes should be made public no later than three (3) years after the end of data collection. Authorship for abstracts and manuscripts resulting from this study will be determined according to guidelines established by the International Committee of Medical Journal Editors.

16 REFERENCES

American Cancer Society. Cancer Facts & Figures 2008. http://www.cancer.org/downloads/STT/2008CAFFfinalsecured.pdf

American Society of Clinical Oncology (ASCO), Kris MG, Hesketh PJ, Somerfield MR, Feyer P, Clark-Snow R, et al. American Society of Clinical Oncology guideline for antiemetics in oncology: update 2006. J Clin Oncol. 2006; 24(18):2932-47. Erratum in: J Clin Oncol. 2006;24(33):5341-2.

Benson AB 3rd, Ajani JA, Catalano RB, Engelking C, Kornblau SM, Martenson JA Jr, et al. Recommended guidelines for the treatment of cancer treatment-induced diarrhea. J Clin Oncol. 2004 Jul 15; 22(14):2918-26.

Bottaro DP, Liotta LA. Out of air is not out of action. Nature. 2003;423:593-5.

Brown JE, Cook RJ, Major P, et al. Bone turnover markers as predictors of skeletal complications in prostate cancer, lung cancer, and other solid tumors. JNCI 2005; 97: 59-69.

Brown JE and Sim S. Evolving role of bone biomarkers in castration-resistant prostate cancer. Neoplasia 2010; 12: 685-96.

Buckanovich RJ, Berger R, Sella A, Sikic BI, Shen X, Ramies DA, et al. Activity of Cabozantinib (XL184) in Advanced Ovarian Cancer Patients: Results From a Phase 2 Randomized Discontinuation Trial (RDT). ASCO Meeting Abstracts 2011;5008.

Cleeland CS, Mendoza TR, Want XS. Assessing symptom distress in cancer patients. The MD Anderson Symptom Inventory. Cancer 2000; 89: 1634-46.

Coleman RE. Skeletal complications of malignancy. Cancer 1997; 80: 1588-94.

Coleman RE, Major P, Lipton A, et al. Predictive value of bone resorption and formation markers in cancer patients with bone metastases receiving the bisphosphonate zoledronic acid. JCO 2005; 23: 4925-35.

Cook RJ, Coleman R, Brown J, et al. Markers of bone metabolism and survival in men with hormone-refractory metastatic prostate cancer. Clin Cancer Res 2006; 12: 3361-7.

Demers LM, Costa L, Lipton A. Biochemical markers and skeletal metastases. Cancer 2000; 88:2919-26.

Eder JP, Heath E, Appleman L, Shapiro G, Wang D, Malburg L, et al. Phase I experience with c-MET inhibitor XL880 administered orally to patients (pts) with solid tumors. 2007 ASCO Annual Meeting Proceedings Part I. J Clin Oncol. 2007;25:18S (June 20 Supplement) (Abstract 3526).

Garcia A, Rosen L, Cunningham CC, Nemunaitis J, Li C, Rulewski N, et al. Phase 1 study of ARQ 197, a selective inhibitor of the c-Met RTK in patients with metastatic solid tumors reaches recommended phase 2 dose. 2007 ASCO Annual Meeting Proceedings Part I. J Clin Oncol. 2007;25:18S (June 20 Supplement) (Abstract 3525).

Gordon MS, Vogelzang NJ, Schoffski P, Daud A, Spira I, O'Keeffe BA, et al. Activity of cabozantinib (XL184) in soft tissue and bone: Results of a phase II randomized discontinuation trial (RDT) in patients (pts) with advanced solid tumors. ASCO Meeting Abstracts 2011; 3010.

Grano M, Galimi F, Zambonin G, et al. Hepatocyte growth factor is a coupling factor for osteoclasts and osteoblasts in vitro. Proc Natl Acad Sci U S A. 1996;93(15):7644-8.

Guise TA, Mohammad KS, Clines G. Basic mechanisms responsible for osteolytic and osteoblastic bone metastases. Clin Cancer Res 2006; 12: (20 Suppl) 6213s-6s.

Hamaoka T, Madewell JE, Podoloff DA, et al. Bone Imaging in Metastatic Breast Cancer. JCO 2004; *22:2942-2953.*

Hesketh PJ. Chemotherapy-induced nausea and vomiting. NEJM. 2008;358(23):2482-94.

Hu EY, Wilding G, Posadas E, et al. Phase II study of dastatinib in patients with metastatic castration-resistanct prostate cancer. Clin Cancer Res 2009; 15: 7421-8.

Humphrey PA, Zu X, Zarnegar R, et al. Hepatocyte growth factor and HS receptor (c-Met) in prostatic carcinoma. American Journal of Pathology 1995; 147(2): 386-396.

Hurwitz H, Fehrenbacher L, Novotny W, Cartwright T, Hainsworth J, Heim W, et al. Bevacizumab plus Irinotecan, Fluorouracil, and Leucovorin for metastatic colorectal cancer. New Engl J Med. 2004;350(23):2335-42.

Hussain M, Smith MR, Sweeney C, Corn PG, Elfiky A, Gordon MS, et al. Cabozantinib (XL184) in metastatic castration-resistant prostate cancer (mCRPC): Results from a phase II randomized discontinuation trial. ASCO Meeting Abstracts 2011; 4516.

Inaba M, Koyama H, Hino M, et al. Regulation of release of hepatocyte growth factor from human promyelocytic leukemia cells, HL-60, by 1,25-dihydroxyvitamin D3, 12-O-tetradecanoylphorbol 13-acetate, and dibutyryl cyclic adenosine monophosphate. Blood. 1993; 82(1):53-9.

Jiang WG, Martin TA, Parr C, Davies G, Matsumoto K, Nakamura T. Hepatocyte growth factor, its receptor, and their potential value in cancer therapies, Crit Rev Oncol Hematol. 2005; 53(1):35-69.

Knudsen BS, Gmyrek GA, Inra J, et al. High expression of the Met receptor in prostate cancer metastasis to bone. Urology 2002; 60: 1113-1117.

Kurzrock R, Sherman SI, Ball DW, Forastiere AA, Cohen RB, et al. Activity of XL184 (Cabozantinib), an Oral Tyrosine Kinase Inhibitor, in Patients With Medullary Thyroid Cancer. J Clin Oncol. 2011; 29(19):2660-6.

Leonardi R, Caltabiano R, Loreto C. The immunolocalization and possible role of c-Met (MET, hepatic growth factor receptor) in the developing human fetal mandibular condyle. Acta Histochem. 2010; 112(5):482-8. Epub 2009 Jun 18.

Lipton A, Cook R, Saad F. Normalization of bone markers is associated with improved survival in patients with bone metastases from solid tumors and elevated bone resorption recevigin zoledronic acid. Cancer 2008; 113: 193-201.

Macdonald DR, Cascino TL, Schold SC Jr, Cairncross JG. Response criteria for Phase 2 studies of supratentorial malignant glioma. J Clin Oncol. 1990;8:1277–80.

Mundy GR. Mechanisms of bone metastasis. Cancer 1997; 80: 1546-56.

Mundy GR. Metastsis to bone: causes, consequences, and therapeutic opportunities. Nature Rev Cancer 2002; 2: 584-93.

Ono K, Kamiya S, Akatsu T, et al. Involvement of hepatocyte growth factor in the development of bone metastasis of a mouse mammary cancer cell line, BALB/c-MC. Bone. 2006; 39(1):27-34. Epub 2006 Feb 3.

Osoba D, Rodrigues G, Myles J, et al. Interpreting the significance of changes in health-related quality-of-life scores. JCO 1998; 16: 139-44.

Pennacchietti S, Michieli P, Galluzzo M, Mazzone M, Giordano S, Comoglio PM. Hypoxia promotes invasive growth by transcriptional activation of the Met protooncogene. Cancer Cell. 2003; 3:347-61.

Pisters LL, Troncoso P, Zhau HE, et al. c-met Proto-oncogene expression in benign and malignant human prostate tissues. J Urol. 1995; 154(1):293-8.

Roila F, Herrstedt J, Aapro M, Gralla RJ, Einhorn LH, Ballatori E, et al. Guideline update for MASCC and ESMO in the prevention of chemotherapy- and radiotherapy-induced nausea and vomiting: results of the Perugia consensus conference. Ann Oncol. 2010; 21 Suppl 5:v232-43.

Rodriguez LAG, Hernández-Díaz S, et al. The risk of upper gastrointestinal complications associated with nonsteroidal anti-inflammatory drugs, glucocorticoids, acetaminophen, and combinations of these agents. Arthritis Res. 2001; 3(2):98–101.

Rosen LS, Gordon D, Tchekmedyian S, et al. Zoledronic Acid Versus Placebo in the Treatment of Skeletal Metastases in Patients With Lung Cancer and Other Solid Tumors: A Phase III, Double-Blind, Randomized Trial—The Zoledronic Acid Lung Cancer and Other Solid Tumors Study Group. JCO 2003; 21:3150-3157.

Ross RW, Stein M, Sarantopoulos J, Eisenberg P, Logan T, Srinivas S, et al. A phase II study of the c-Met RTK inhibitor XL880 in patients (pts) with papillary renal-cell carcinoma (PRC). 2007 ASCO Annual Meeting Proceedings Part I. J Clin Oncol. 2007; 25:18S (June 20 Supplement) (Abstract 15601).

Sandler AB, Gray R, Brahmer J, Dowlati A, Schiller JH, Perry MC, et al, Randomized phase II/III Trial of paclitaxel (P) plus carboplatin (C) with or without bevacizumab (NSC #704865) in patients with advanced non-squamous non-small cell lung cancer (NSCLC): An Eastern Cooperative Oncology Group (ECOG) Trial - E4599. ASCO 2005 Annual Meeting (Abstract LBA4).

Sattler M, Ma PC, Salgia R. Therapeutic targeting of the receptor tyrosine kinase Met. Cancer Treat Res. 2004; 119:121-38.

Straube S, Tramèr MR, et al. Mortality with upper gastrointestinal bleeding and perforation: effects of time and NSAID use. BMC Gastroenterol. 2009;9:41.

Street J and Lenehan B. Vascular endothelial growth factor regulates osteoblast survival – evidence for an autocrine feedback mechanism. J Orthop Surg Res. 2009; 4:19.

Tolaney S, Nechustan H, Berger R et al. Cabozantinib (XL184) in Patients With Metastatic Breast Cancer: Results From a Phase 2 Randomized Discontinuation Trial. San Antonio Breast Cancer Symposium 2011.

Wright JR, Ung YC, Julian JA, Pritchard KI, Whelan TJ, Smith C, et al. Randomized, double-blind, placebo-controlled trial of erythropoietin in non-small-cell lung cancer with disease-related anemia. J Clin Oncol. 2007; 25:1027-32.

Zelzer E and Olsen BR. Multiple roles of vascular endothelial growth factor (VEGF) in skeletal development, growth, and repair. Curr Top Dev Biol. 2005; 65:169-87.

Zhang S, Zhau H, Osunkoya A, et al. Vascular endothelial growth factor regulates myeloid cell leukemia-1 expression through neuropilin-1-dependent activation of c-MET signaling in human prostate cancer cells. Mol Cancer. 2010; 9:9.

APPENDIX A: PERFORMANCE STATUS CRITERIA

ECOG Performance Status Scale		Karnofsky Performance Scale	
Grade	Descriptions	Percent	Description
0	Normal activity. Fully active, able to carry on all predisease performance without restriction.	100	Normal, no complaints, no evidence of disease.
		90	Able to carry on normal activity; minor signs or symptoms of disease.
1	Symptoms, but ambulatory. Restricted in physically strenuous activity, but ambulatory and able to carry out work of a light or sedentary nature (eg, light housework, office work).	80	Normal activity with effort; some signs or symptoms of disease.
		70	Cares for self, unable to carry on normal activity or to do active work.
2	In bed < 50% of the time. Ambulatory and capable of all self-care, but unable to carry out any work activities. Up and about more than 50% of waking hours.	60	Requires occasional assistance, but is able to care for most of his/ her needs.
		50	Requires considerable assistance and frequent medical care.
3	In bed > 50% of the time. Capable of only limited self-care, confined to bed or chair more than 50% of waking hours.	40	Disabled, requires special care and assistance.
		30	Severely disabled, hospitalization indicated. Death not imminent.
4	100% bedridden. Completely disabled. Cannot carry on any self-care. Totally confined to bed or chair.	20	Very sick, hospitalization indicated. Death not imminent.
		10	Moribund, fatal processes progressing rapidly.
5	Dead	0	Dead

ECOG = Eastern Cooperative Oncology Group.

APPENDIX B: CABOZANTINIB TABLET COMPONENTS AND COMPOSITION

Table B-1: Cabozantinib Tablet Components and Composition

Ingredient	Function	% w/w
XL-184 Drug Substance (25% drug load as free base)	Active Ingredient	31.7
Microcrystalline Cellulose (Avicel PH-102)	Filler	38.9
Lactose Anhydrous (60M)	Filler	19.4
Hydroxypropyl Cellulose (EXF)	Binder	3.0
Croscarmellose Sodium (Ac-Di-Sol)	Disenegrant	6.0
Colloidal Silicon Dioxide,	Glidant	0.3
Magnesium Stearate	Lubricant	0.75
Opadry Yellow Film Coating which includes:<br>- HPMC 2910 / Hypromellose 6 cp<br>- Titanium dioxide<br>- Triacetin<br>- Iron Oxide Yellow	Film Coating	4.00

Any unused cabozantinib must be returned to the study site for disposal.
